# Targeting USP11 counteracts *SFTPC^I73T^*-associated interstitial lung disease in hiPSCs-derived alveolar organoids and in vivo models

**DOI:** 10.7150/thno.105994

**Published:** 2025-03-19

**Authors:** Janardhan Keshav Karapurkar, Sripriya Rajkumar, Ji-Hye Jung, Ji-Young Kim, Girish Birappa, D. A. Ayush Gowda, Jencia Carminha Colaco, Bharathi Suresh, Jung-Yun Choi, Sang Hyeon Woo, Won-Jun Jo, Jong-Hee Lee, Kye-Seong Kim, Seok-Ho Hong, Suresh Ramakrishna

**Affiliations:** 1 Graduate School of Biomedical Science and Engineering, Hanyang University, Seoul, 04763, South Korea.; 2 Department of Internal Medicine, College of Medicine, Kangwon National University, Chuncheon, South Korea.; 3 National Primate Research Center (NPRC), Korea Research Institute of Bioscience and Biotechnology (KRIBB), Cheongju, 28116, South Korea.; 4 KW-Bio Co., Ltd, Chuncheon, South Korea.; 5 College of Medicine, Hanyang University, Seoul, 04763, South Korea.

**Keywords:** alveolar organoid, BLM-induced fibrosis, mutant variants, mitoxantrone, pulmonary fibrosis, protein turnover, protein misfolding, protein accumulation, therapeutics, TGF-β-induced fibrosis.

## Abstract

**Background:** Interstitial lung disease (ILD) is a pulmonary disorder characterized by a combination of inflammation and fibrosis in the lung parenchyma, which initiates with the dysfunction of alveolar epithelial cells (AECs). The alveolar cells secrete surfactant proteins that lowers the surface tension of fluids in the lungs and maintains the stability of pulmonary tissue. Mutations on surfactant protein C (SFTPC), particularly I73T, are associated with a toxic gain of function that causes misfolding and the accumulation of immature SFTPC proteins, triggering pulmonary fibrosis (PF). Therefore, it is crucial to block the accumulation of the SFTPC*^I73T^* protein during ILD progression.

**Methods:** We used a loss-of-function-based CRISPR/Cas9 library kit to screen genome-wide for deubiquitinating enzymes that regulate the SFTPC protein. The interaction between USP11 and SFTPC and its ubiquitination status was validated by immunoprecipitation and the TUBEs assay. HDR-directed knock-in of the I73T mutation into the *SFTPC* locus in human induced pluripotent stem cells (hiPSCs) was performed using the CRISPR/Cas9 system, and then those cells were differentiated into alveolar organoids (AOs) using a forced aggregation protocol. The clinical relevance of the USP11 inhibitor and its effect on preventing PF were investigated in a TGF-β-induced fibrosis in AOs and bleomycin (BLM)-induced mouse model.

**Results:** We identified USP11 as a novel deubiquitinase that interacts with, stabilizes, deubiquitinates, and extends the half-life of SFTPC. Remarkably, USP11 stabilized and prolonged the half-life of the SFTPC*^I73T^* mutant protein significantly more than the wild type. In vitro functional studies revealed that USP11 exacerbates SFTPC*^I73T^*-induced fibrosis and enhances the epithelial-to-mesenchymal transition. Furthermore, we present a human in vitro model for investigating *SFTPC^I73T^*-induced fibrosis: hiPSCs-derived-AOs carrying the pathogenic *SFTPC^I73T^* variant. Interestingly, USP11 depletion in the organoids mitigated SFTPC*^I73T^*-induced fibrosis. Finally, pharmacological inhibition of USP11 prevented PF caused by TGF-β in hiPSCs-SFTPC^I73T^-AOs and BLM-induced mouse model, underscoring its therapeutic potential.

**Conclusions:** Altogether, USP11 is a major protein stabilizer of SFTPC, and the clinical inhibition of USP11 during PF could be a novel therapeutic approach for ILD patients.

## Introduction

Interstitial lung disease (ILD) is a progressive and frequently fatal lung disease, characterized by the steady accumulation of fibrotic tissue within the lungs, which eventually impairs lung function and causes respiratory failure 1. Idiopathic pulmonary fibrosis (IPF) is one of the disease's manifestations, though its exact cause is unknown. IPF causes pathological scarring of the lung that produces stiffness and makes gas exchange difficult, eventually leading to premature death 2, 3. The development of IPF depends on alveolar type 2 (AT2) epithelial cells, which are the precursors of the AT1 cells responsible for the synthesis of surfactant 4. It is increasingly acknowledged that pathological lung remodeling in IPF is caused by AT2 injury and dysfunction 5.

Among the several types of lung epithelia, malfunction of the AT2 epithelial cell was considered critical in the pathogenesis of ILD 6. However, it has proven difficult to identify the exact mechanism by which AT2 dysfunction causes disease in humans 7, 8. Recently, several reports suggested that etiological factors such as drugs, environmental exposures, or genetic mutations in AT2-specific genes play a role in the development of ILD 9. Because the disease-associated variant of the *surfactant protein C* (*SFTPC*) gene expresses exclusively in AT2 10, we here focus on autosomal dominant mutations in *SFTPC* and its post-translational regulation during the pathogenesis of ILD.

The *SFTPC* gene is responsible for maintaining alveolar stability by reducing surface tension at the air-liquid interface in the lungs, preventing alveolar collapse, and facilitating efficient gas exchange. This equilibrium can be altered by mutations in the *SFTPC* gene that cause surfactant malfunction and eventually lead to PF. Among several reported mutations of *SFTPC*, the I73T mutation is the most predominant and pathogenic. I73T is a missense mutation, g.1286T>C, that replaces isoleucine with threonine at the 73rd amino acid, resulting in a toxic gain of function 11. The I73T mutation on *SFTPC* causes misfolding of the SFTPC protein, which then accumulates within AECs, delaying its protein trafficking and turnover 12.

To uncover the abundance of the pathogenic SFTPC*^I73T^* protein in ILD, we must understand SFTPC protein turnover. Pro-SFTPC, the precursor form of SFTPC, is synthesized and passed through multiple proteolytic stages prior to secretion. Human SFTPC is synthesized in AT2 cells as a pro-protein that later undergoes endoproteolytic C- and N-terminal cleavages to yield mature SFTPC 13-15. Any mutations in SFTPC, particularly the I73T mutation, cause aberrant biosynthesis of the mature SFTPC protein, which causes an abnormal abundance of misprocessed pro-SFTPC at the plasma membrane 16, 17. In other words, inefficient post-translational processing and turnover of immature pro-SFTPC has toxic effects on AT2 function. The ubiquitin proteasomal system plays an important role in the regulation of misfolded protein turnover. Thus, understanding this mechanism enables us to develop novel therapeutics to prevent the accumulation of immature pro-SFTPC and the onset of fibrosis 10.

Deubiquitinating enzymes (DUBs) are crucial for the progression of several diseases because they can counteract ubiquitination by cleaving ubiquitin molecules from disease-related proteins targeted for proteolysis 18. Ubiquitin-specific proteases (USPs) are the largest subfamily of DUBs; they remove ubiquitin molecules from their targeted proteins and are involved in the regulation of several diseases, including lung diseases 19, 20. Recently, DUBs have emerged as the main stabilizers of key proteins involved in the development of fibrosis in the heart, kidney, liver, and lungs. In particular, USP13, USP18, USP38, and A20 are involved in the progression of PF 21-23.

In this study, we applied our recently developed CRISPR-based single-guide RNA (sgRNA) library targeting DUBs 24-26 to screen for novel DUBs that might stabilize the abundance of the SFTPC protein. We identified USP11 as a regulator of SFTPC that extends its protein half-life. Additionally, we demonstrated that USP11 has strong deubiquitinating and stabilizing effects on the SFTPC*^I73T^* mutant protein. To understand the pathogenesis of SFTPC*^I73T^*-related PF, we used advanced alveolar organoids (AOs) derived from human induced pluripotent stem cells (hiPSCs) carrying the *SFTPC^I73T^* mutation. AOs are 3D cell culture models that mimic the structure and functions of the proximal and distal airways and alveoli 27. This model system allowed us to demonstrate that the spontaneous fibrotic changes caused by the *SFTPC^I73T^* mutation were significantly attenuated by the knockdown of USP11. Finally, we demonstrated that the pharmacological inhibition of USP11 prevented PF caused by TGF-β in hiPSCs-SFTPC^I73T^-AOs and bleomycin-induced pulmonary fibrosis (BLM-IPF) mouse model. Therefore, we envision that targeting USP11 may provide a novel therapeutic approach to counter PF by regulating SFTPC protein stability.

## Results

### Genome-scale screening of DUBs that regulate SFTPC protein abundance

**Figure 1:**
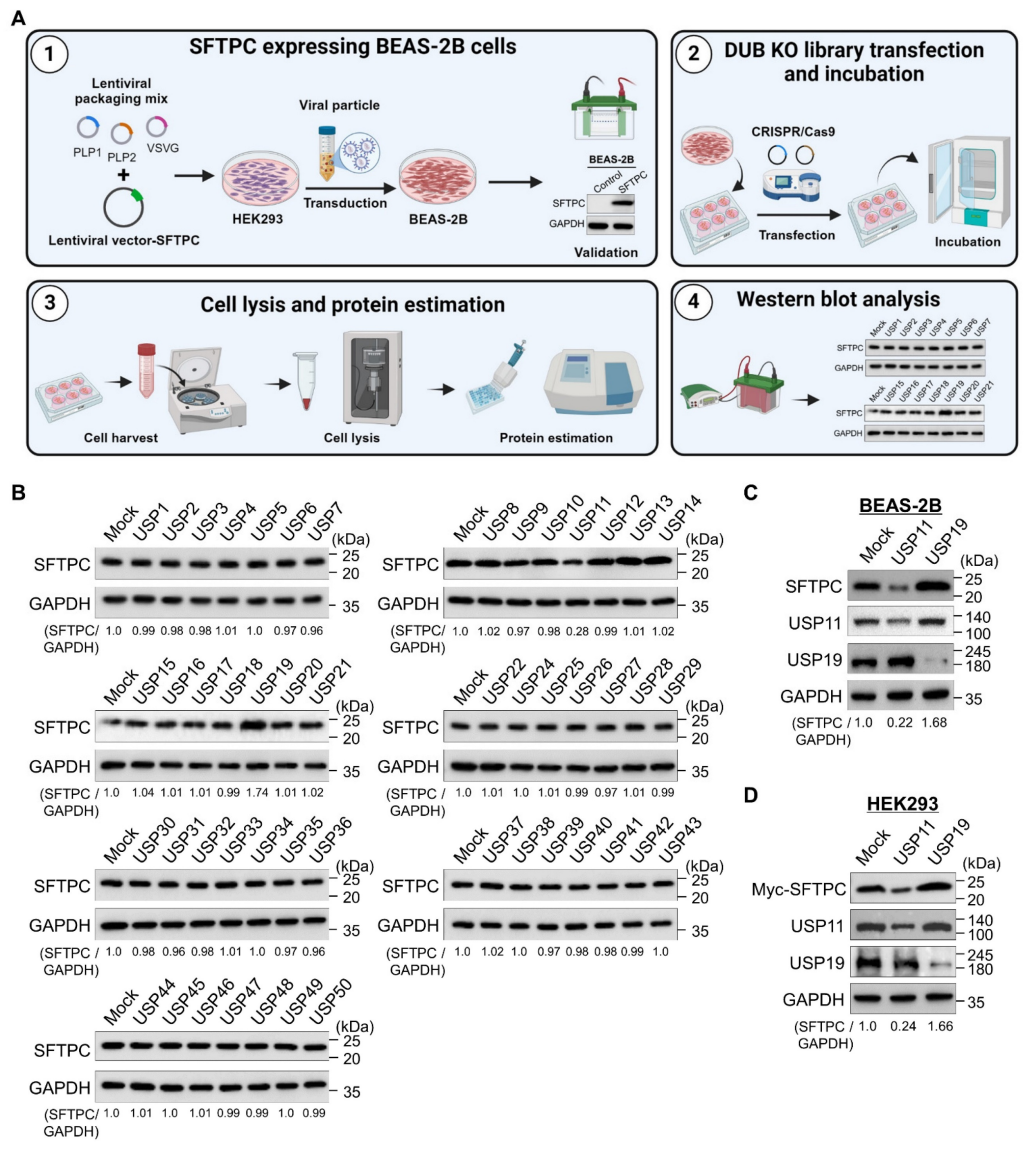
**CRISPR/Cas9-based genome-scale screening of USP subfamily proteins regulating SFTPC protein level. (A)** DUB knockout (KO) library screening system. Step 1. Establish BEAS-2B cell lines stably expressing the SFTPC protein. HEK293 cells were transfected with a lentiviral vector encoding the *SFTPC* gene and packaging constructs. The viral particles were transduced into BEAS-2B cells, and stable expression of the SFTPC protein was checked by western blot. Step 2. BEAS-2B-SFTPC cells were seeded in 6-well plates at a density of 3.5 X 10^5^ cells/well and maintained in DMEM. Step 3. The DUB KO library, consisting of sgRNAs individually targeting an entire set of USP family members, were co-transfected with Cas9 into the BEAS-2B-SFTPC cells using Lipofectamine 3000. Step 4. The transfected cells were maintained in DMEM containing puromycin (1.5 mg/mL) for 72 h and then harvested. Step 5. Western blotting was performed using the SFTPC antibody to identify DUBs regulating the stability of SFTPC protein levels. **(B)** Equal protein concentrations from the cell lysates from **(A)** were subjected to western blotting to determine the endogenous SFTPC protein level. For each blot, BEAS-2B-SFTPC cells co-transfected with scrambled sgRNA and Cas9 served as the mock control. GAPDH was used as the loading control. The protein band intensities were estimated using ImageJ software with reference to the GAPDH control for each individual sgRNA (SFTPC/GAPDH) and are presented below each blot. **(C-D)** Western blot analyses of putative DUB candidates' effects on the **(C)** endogenous SFTPC and **(D)** exogenous SFTPC protein levels. The protein band intensities were estimated using ImageJ software with reference to the GAPDH control band for each individual sgRNA (SFTPC/GAPDH) and are presented below each blot.

To identify DUBs that regulate the SFTPC protein level, we used a recently developed CRISPR/Cas9-based loss-of-function DUB-knockout library kit 24, 28. For this purpose, we generated BEAS-2B cell lines that stably expressed the SFTPC protein (BEAS-2B-SFTPC^WT^ cells) using an integrated lentiviral vector (Figure 1A). The DUB knockout library contains sgRNAs that target entire genes belonging to the USP subfamily. These sgRNAs were co-transfected with Cas9 into the BEAS-2B-SFTPC^WT^ cells. Alterations in SFTPC protein levels were analyzed by western blotting (Figure 1A). The decrease in SFTPC protein abundance caused due to the loss of function of a specific DUBs were considered as putative positive candidates for further analysis. Our screening results indicated that the loss of USP11 decreased SFTPC protein levels when compared with the mock control, while the loss of USP19 increased SFTPC expression (Figure 1B). We cross-confirmed the effects of loss of USP11 and USP19 on endogenous SFTPC protein levels in BEAS-2B-SFTPC^WT^ cells (Figure 1C) and exogenous SFTPC protein levels in HEK293 cells (Figure 1D). Our results show that USP11 depletion significantly decreased both endogenous and ectopic SFTPC protein (Figure 1C and 1D). Mutations in the *SFTPC* gene alter its protein biosynthesis, causing the misprocessed SFTPC protein to accumulate and resulting in AT2 dysfunction 10. Therefore, we investigated the function of the USP11-mediated SFTPC protein abundance during lung fibrosis.

### USP11 increases SFTPC protein levels

**Figure 2:**
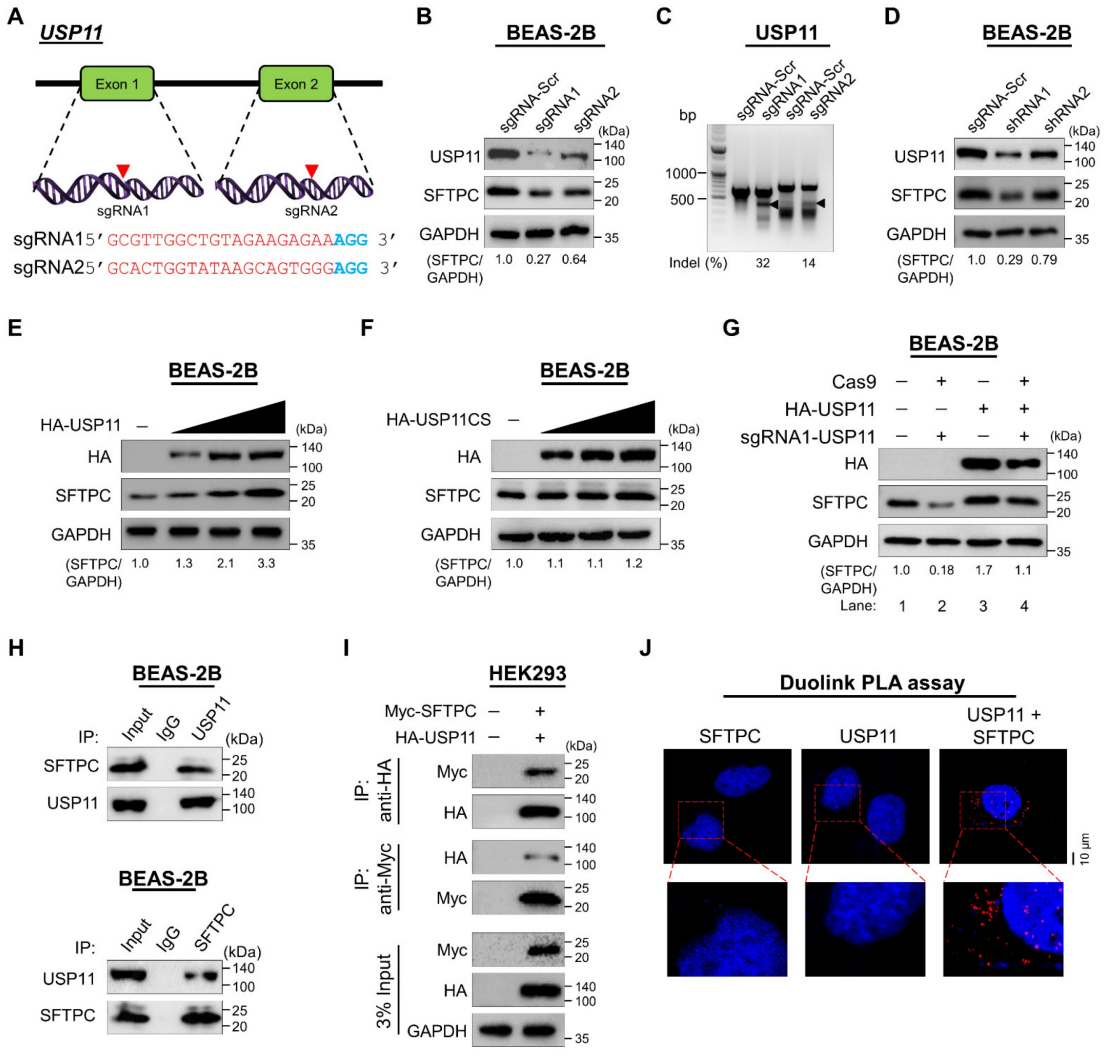
**USP11 stabilizes and interacts with the SFTPC protein. (A)** Is a schematic representation of the sgRNAs targeting exons 1 and 2 of the USP11 gene. Red arrowheads indicate the positions of the sgRNAs targeting the top strand. sgRNA sequences are in red; PAM sequences are in bold blue. **(B)** The efficiency of sgRNAs targeting USP11 was validated by western blot using the USP11 antibody, and their effects on SFTPC were tested using the SFTPC antibody. **(C)** Validation of sgRNA efficiency in targeting the USP11 gene by transiently co-transfecting BEAS-2B cells with Cas9 and sgRNA1 or sgRNA2, followed by a T7E1 assay to determine cleavage efficiency. The cleaved band intensity (indel %) obtained in the T7E1 assay was measured using ImageJ software and is indicated below each blot. BEAS-2B cells transfected with scrambled sgRNA were used as controls. The black arrowhead indicates cleaved PCR amplicons. **(D)** BEAS-2B cells were transfected with shRNAs targeting USP11, and the endogenous protein levels of USP11 and SFTPC were checked by western blot. **(E-F)** BEAS-2B cells were transfected with increasing concentrations of **(E)** HA-USP11 or **(F)** HA-USP11CS to check the endogenous SFTPC protein levels. **(G)** The reconstitution effect of HA-USP11 on endogenous SFTPC protein in USP11-depleted BEAS-2B cells. The protein band intensities were estimated using ImageJ software with reference to the GAPDH control band (SFTPC/GAPDH) and are presented below each blot. **(H)** Interactions between endogenous and **(I)** exogenous USP11 and SFTPC proteins were analyzed in BEAS-2B and HEK293 cells, respectively. Cell lysates were immunoprecipitated and immunoblotted with the indicated antibodies. Protein expression was checked using western blotting. GAPDH was used as a loading control. **(J)** BEAS-2B cells were subjected to the Duolink PLA assay to analyze the interaction between USP11 and SFTPC using specific antibodies. *In situ* USP11-SFTPC interactions (PLA dots) were imaged through microscopy. Scale bar: 10 μm.

To validate the stabilizing effect of USP11 on SFTPC protein levels, we applied both sgRNAs and short hairpin RNA (shRNAs) targeting *USP11* to deplete its expression. The sgRNAs were designed to target exon 1 and exon 2 of the *USP11* gene (Figure 2A). Transient transfection of sgRNA1 targeting *USP11* produced a significantly larger decrease in USP11 expression than transfection with sgRNA2 (Figure 2B; upper panel), which is in line with the higher indel percentage observed in sgRNA1, compared with sgRNA2 (Figure 2C). Moreover, cells transfected with sgRNA1 targeting USP11 exhibited a significant reduction in their SFTPC protein levels (Figure 2B; middle panel). Likewise, transfection with shRNA1 targeting *USP11* produced greater reductions in both USP11 and SFTPC protein levels than shRNA2 (Figure 2D). Therefore, we used sgRNA1 (hereafter sgRNA) and shRNA1 (hereafter shRNA) to further investigate the role of USP11 depletion in regulating SFTPC protein levels.

Next, we transfected BEAS-2B-SFTPC^WT^ cells with increasing concentrations of HA-USP11 and a catalytically inactive mutant HA-USP11 with a cysteine to serine mutation at position 318 (hereafter HA-USP11CS) to analyze the SFTPC protein levels. The dose-dependent transfection of HA-USP11 showed a substantial increase of SFTPC protein level (Figure 2E), whereas the transfection of HA-USP11CS did not affect the SFTPC protein level (Figure 2F). Furthermore, the reduced expression of SFTPC protein in USP11-depleted cells (Figure 2G; lane 2) was rescued by transient transfection with HA-USP11 (Figure 2G; lane 4). These results indicate that USP11 positively regulates SFTPC protein levels.

### USP11 interacts with SFTPC protein

Next, to explain the mechanism by which USP11 regulates the SFTPC protein level, we examined whether USP11 physically associates with SFTPC in vivo by using a co-immunoprecipitation (co-IP) assay with specific antibodies against endogenous USP11 and SFTPC. We demonstrated that USP11 and the SFTPC protein interact endogenously with each other in physiological conditions (Figure 2H). Additionally, HA antibody against ectopically expressed HA-USP11 co-immunoprecipitated with Myc-SFTPC (Figure 2I; upper panel) and vice versa (Figure 2I; lower panel).

Furthermore, we used Duolink PLA analysis, wherein the fluorescent signals (PLA dots) represent interactions between the target and substrate, to visualize *in situ* interactions between endogenous USP11 and SFTPC 29. *In situ* interactions between USP11 and SFTPC were confirmed by PLA dots showing where USP11 and SFTPC were immunostained together in the presence of their specific antibodies (Figure 2J; right panel). However, no PLA dots were observed in cells stained with either USP11 or SFTPC antibody alone (Figure 2J; left and middle panel), suggesting that USP11 interacts with the SFTPC protein endogenously.

### The deubiquitinating activity of USP11 prevents SFTPC protein degradation

**Figure 3:**
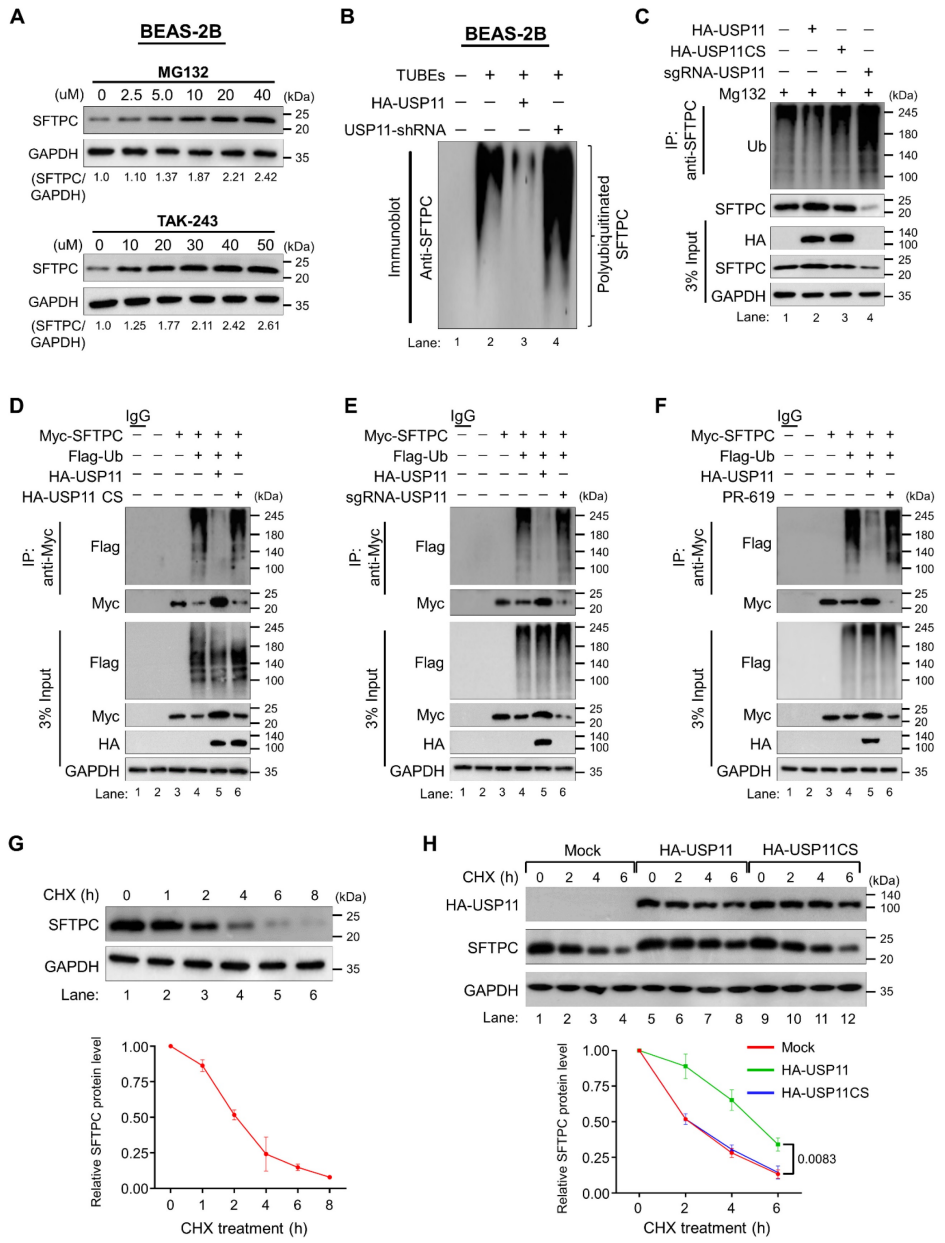
**USP11 deubiquitinates SFTPC and extends its half-life. (A)** BEAS-2B cells were treated with the indicated concentrations of a proteasomal inhibitor (MG132) or an inhibitor of ubiquitin activating enzyme (TAK-243) for 6 h prior to harvest. Immunoblotting was performed with the indicated antibodies. The protein band intensities were estimated using ImageJ software with reference to the GAPDH control band (SFTPC/GAPDH) and are presented below the blots. **(B)** BEAS-2B cells were transfected with HA-USP11 or HA-USP11-shRNA and treated with MG132 for 6 h prior to harvest. A TUBEs assay was performed to assess the ubiquitination status of the SFTPC protein in mock control and HA-USP11 or HA-USP11-shRNA transduced cells. Cell lysates were immunoprecipitated with TUBEs antibodies, followed by immunoblotting with the indicated antibodies. **(C)** The ubiquitination and deubiquitination of endogenous SFTPC were analyzed by transfecting BEAS-2B cells with HA-USP11, HA-USP11CS, or sgRNA targeting USP11 followed by immunoprecipitation (IP) with an anti-SFTPC antibody and immunoblotting with an anti-ubiquitin antibody. The cells were treated with MG132 for 6 h prior to harvest. **(D-F)** The ubiquitination and deubiquitination of ectopically expressed Myc-SFTPC were analyzed by transfecting HEK293 cells with Flag-Ub along with **(D)** HA-USP11 and HA-USP11CS **(E)** HA-USP11 and sgRNA targeting USP11 **(F)** treatment of DUB-inhibitor PR-619 for 48 h in the HEK293 cell line prior to harvest, followed by IP with a Myc antibody and immunoblotting with an anti-Flag antibody. The relative protein expression of SFTPC-(Ub)n with respect to the input SFTPC for **(C-F)** was quantified using ImageJ software and is represented as (SFTPC-(Ub)n/SFTPC) below each blot. **(G)** The half-life of endogenous SFTPC in BEAS-2B cells. **(H)** The half-life of endogenous SFTPC in the presence of HA-USP11 or HA-USP11CS in BEAS-2B cells. CHX (250 μg/mL) was administered for the indicated time, and the cells were then harvested for western blotting with the indicated antibodies. Data are presented as the mean and standard deviation of three independent experiments (n = 3). Two-way ANOVA followed by Tukey's post hoc test was used with the indicated *P*-values.

To determine whether the SFTPC protein undergoes degradation via the 26S proteasomal degradation pathway, we treated cells with either MG132, a proteasome inhibitor, or TAK-243, an inhibitor of the ubiquitin-activating enzyme. Treatment with increasing concentrations of both MG132 and TAK-243 increased the SFTPC protein level dose dependently, suggesting that SFTPC protein turnover is regulated by the proteasomal degradation pathway (Figure 3A).

To further explore the status of SFTPC ubiquitination and the mechanism by which USP11 regulates SFTPC protein turnover, we assessed the deubiquitinating activity of USP11 on endogenous SFTPC protein in BEAS-2B-SFTPC^WT^ cells. The tandem ubiquitin binding entities (TUBEs) assay demonstrated that endogenous SFTPC undergoes polyubiquitination (Figure 3B; lane 2).

However, transient expression of USP11 significantly reduced the polyubiquitination smear conjugated with the SFTPC protein (Figure 3B; lane 3). On the contrary, shRNA-mediated knockdown of USP11 markedly increased the polyubiquitination of the SFTPC protein (Figure 3B; lane 4). We verified the deubiquitination activity of USP11 using an IP assay, which showed that USP11 overexpression decreased SFTPC polyubiquitination (Figure 3C; lane 2 vs. lane 1). In contrast, USP11 depletion increased the polyubiquitination of SFTPC (Figure 3C; lane 4 vs. lane 1). On the other hand, the catalytically inactive mutant USP11CS did not have any effect on the deubiquitination of the SFTPC protein (Figure 3C; lane 3).

Consistent with the previous results, the deubiquitination assay on ectopically expressed SFTPC protein in HEK293 cells demonstrated that USP11 removes the polyubiquitin smear conjugated with SFTPC (Figure 3D, E, F; lane 5), and USP11CS did not exhibit any deubiquitinating activity on SFTPC (Figure 3D; lane 6). In contrast, sgRNA-mediated depletion of USP11 expression led to a significant increase in the polyubiquitination smear for the SFTPC protein (Figure 3E; lane 6). Moreover, treatment with a DUB inhibitor, PR619, blocked the deubiquitinating activity of USP11, as evidenced by a significant increase in the polyubiquitination of the SFTPC protein (Figure 3F; lane 6). Overall, those results demonstrate that USP11 prevents the ubiquitin-mediated proteasomal degradation of the SFTPC protein via its deubiquitinating activity.

### USP11 extends the half-life of the SFTPC protein

To analyze SFTPC protein turnover, we estimated its protein half-life. To this end, we treated BEAS-2B-SFTPC^WT^ cells with CHX, a protein synthesis inhibitor, and then checked SFTPC protein expression. CHX treatment resulted in a time-dependent decrease in SFTPC protein levels and showed the half-life of SFTPC to be around 2 h (Figure 3G). To determine the functional consequence of USP11 preventing SFTPC protein degradation, we next checked the effect of USP11 on the half-life of the SFTPC protein. We administered CHX to BEAS-2B-SFTPC^WT^ cells in the presence of USP11 or USP11CS. The protein half-life of SFTPC was significantly extended by USP11 (Figure 3H; lanes 5-8). However, overexpression of the USP11 catalytic mutant did not have any influence on the half-life of the SFTPC protein (Figure 3H; lanes 9-12), suggesting that the deubiquitinating activity of USP11 regulates SFTPC protein turnover.

### The stabilizing effect of USP11 on the pathogenic SFTPC mutant

**Figure 4:**
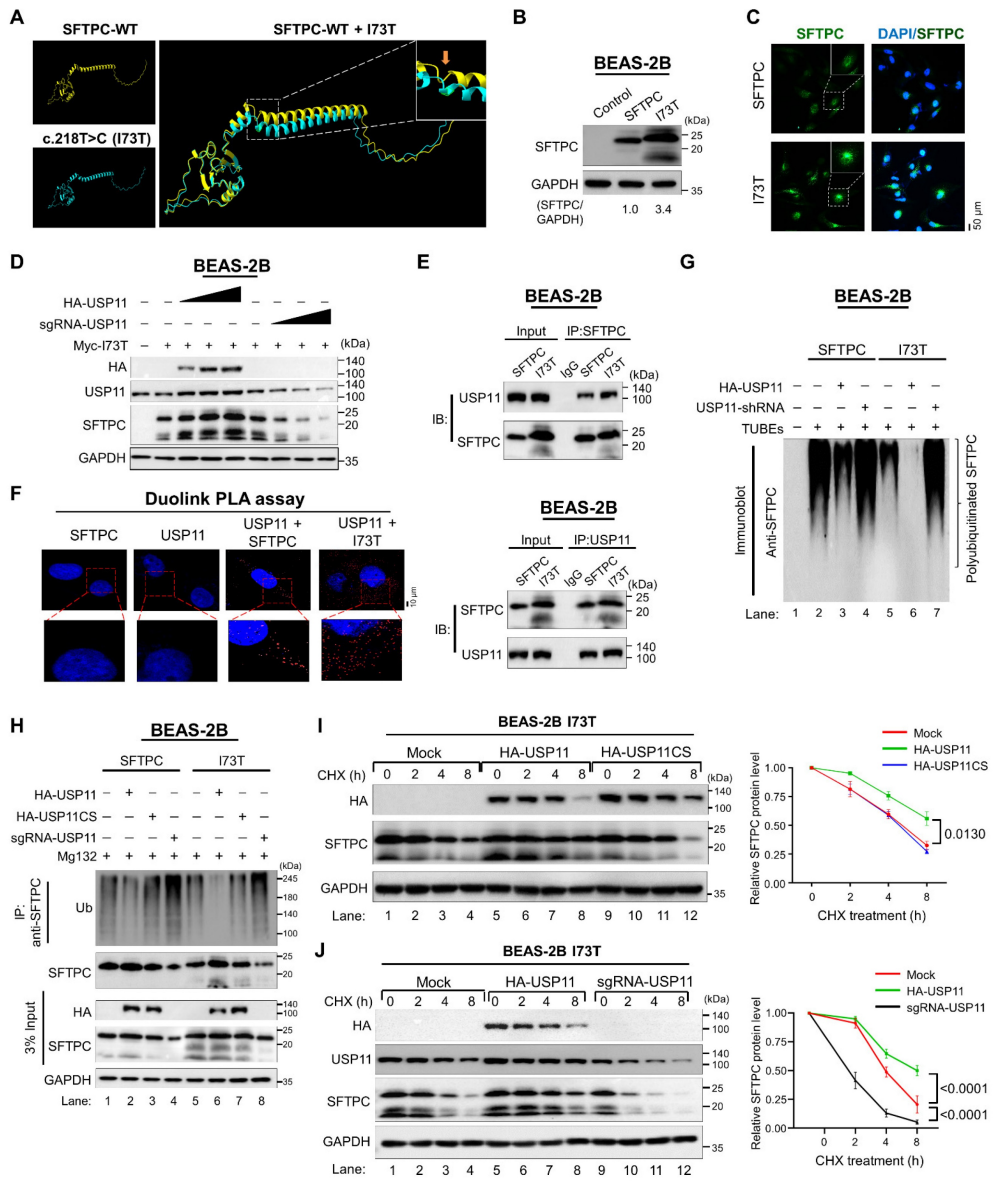
** USP11 deubiquitinates and extends the half-life of the SFTPC*^I73T^* mutant protein. (A)** The predicted 3D structure of wild-type SFTPC and SFTPC*^I73T^* protein was generated using the AlphaFold tool. The structure of wild-type SFTPC was superimposed on that of its mutant at the I73T position using the UCSF chimeraX tool**. (B)** Western blot analysis of the basal level of transduced SFTPC and SFTPC*^I73T^* protein expression. The protein band intensities were estimated using ImageJ software with reference to the GAPDH control band (SFTPC/GAPDH). **(C)** Immunofluorescence analysis of the basal level of transduced SFTPC and SFTPC*^I73T^* protein expression. **(D)** The dose-dependent increasing effect of HA-USP11 and sgRNA-USP11 on SFTPC*^I73T^* protein levels was analyzed by western blotting. **(E)** Interactions between endogenous USP11 and SFTPC proteins were analyzed in BEAS-2B cells. Cell lysates were immunoprecipitated and immunoblotted with the indicated antibodies. **(F)** BEAS-2B-SFTPC WT and BEAS-2B-SFTPC*^I73T^* cells were subjected to the Duolink PLA assay to analyze interactions between USP11 and SFTPC or SFTPC*^I73T^* using specific antibodies. Scale bar: 10 μm. **(G)** The ubiquitination status between the SFTPC and SFTPC*^I73T^* proteins in the presence of HA-USP11 or HA-USP11-shRNA. Cell lysates were immunoprecipitated with TUBEs antibodies, followed by immunoblotting with the indicated antibodies. The cells were treated with MG132 for 6 h prior to harvest. **(H)** The ubiquitination and deubiquitination of endogenous SFTPC and SFTPC*^I73T^* were analyzed in the presence of HA-USP11, HA-USP11CS, or sgRNA targeting USP11, followed by IP with an anti-SFTPC antibody and immunoblotting with an anti-ubiquitin antibody. The cells were treated with MG132 for 6 h prior to harvest. **(I)** The half-life of endogenous SFTPC*^I73T^* in BEAS-2B cells in the presence of HA-USP11 or HA-USP11CS. **(J)** The half-life of endogenous SFTPC*^I73T^* in BEAS-2B cells in the presence of HA-USP11 or sgRNA-USP11. CHX (250 μg/mL) was administered for the indicated time, and the cells were then harvested for western blotting with the indicated antibodies. Data are presented as the mean and standard deviation of three independent experiments (n = 3). Two-way ANOVA followed by Tukey's post hoc test was used with the indicated *P-*values.

Among *SFTPC* variants, the I73T mutation predominantly causes misfolding and accumulation of the SFTPC protein. This mutation impedes protein trafficking and recycling, which affects SFTPC protein turnover 17. Initially, we analyzed the structural difference between wild type SFTPC and SFTPC*^I73T^* using the AlphaFold tool 30, 31. The 3D protein structures of SFTPC and SFTPC*^I73T^* were superimposed to identify the exact position of the I73T mutation. Notably, the 73^rd^ amino acid change from isoleucine to threonine in the SFTPC*^I73T^* protein led to its conformational changes compared with SFTPC-WT (Figure 4A). Next, we set out to determine whether the stabilizing effect of USP11 on the turnover of mutant SFTPC proteins is similar to that on wild-type SFTPC. To this end, we introduced the I73T mutation to the *SFTPC* gene using the site-directed mutagenesis method and confirmed the sequence by Sanger sequencing (Figure S1A). The basal expression level of the mutant SFTPC*^I73T^* was significantly higher than that of wild-type SFTPC (Figure S1B). The mutant SFTPC*^I73T^* was then transduced into BEAS-2B cells to generate a cell line stably expressing the SFTPC*^I73T^* mutation (hereafter BEAS-2B-SFTPC^I73T^) (Figure S2). Consistently, the expression of SFTPC*^I73T^* was greater than that of wild-type SFTPC (Figures 4B and 4C), which was in line with the previous report 10. Additionally, we observed that the stabilizing effect of USP11 was more pronounced on the SFTPC*^I73T^* mutant protein than the SFTPC wild-type protein (Figure S3), but no significant changes were observed with USP11CS (Figure S4), suggesting that USP11 might have higher deubiquitinating activity on the SFTPC*^I73T^* protein than the SFTPC wild-type protein. However, the depletion of USP11 dramatically destabilizes SFTPC*^I73T^* mutant protein, indicating that USP11 is a specific DUB for SFTPC*^I73T^* protein (Figure 4D).

### USP11 interacts with, deubiquitinates, and extends the half-life of the SFTPC*^I73T^* protein

Given the strong stabilizing activity of USP11 on the SFTPC*^I73T^* protein, we wished to examine the binding intensity between them. Co-IP assays using tagged antibodies against ectopically expressed USP11 and SFTPC constructs (Figure S5) and specific antibodies against endogenous USP11 and SFTPC (Figure 4E) demonstrated that the binding intensity between USP11 and SFTPC*^I73T^* is greater than that between USP11 and wild-type SFTPC. This finding was supported by a Duolink PLA analysis that visualized *in situ* interactions between endogenous USP11 and SFTPC or SFTPC*^I73T^*. Significantly more PLA dots were observed for USP11 and SFTPC*^I73T^* than for USP11 and wild-type SFTPC (Figure 4F).

To further investigate the role of USP11 in regulating SFTPC*^I73T^* protein turnover, we performed shRNA-mediated knockdown of USP11 in BEAS-2B-SFTPC^I73T^ cells in the presence and absence of MG132. As expected, USP11 depletion resulted in a significant reduction in the mutant SFTPC*^I73T^* protein levels (Figure S6; lane 2). Conversely, treating USP11-depleted cells with MG132 recovered SFTPC*^I73T^* expression (Figure S6; lane 3), suggesting that SFTPC*^I73T^* protein accumulation is regulated by the ubiquitin-proteasomal system.

Next, we used TUBEs and IP assays to investigate the effect of USP11 on the ubiquitination status of the SFTPC*^I73T^* protein. The SFTPC*^I73T^* mutant protein exhibited a reduced polyubiquitin smear compared with the wild-type SFTPC protein (Figure S7A; lane 3 vs. lane 2). Correspondingly, USP11 exhibited more deubiquitinating activity on the mutant SFTPC*^I73T^* protein than on the wild-type SFTPC (Figure 4G; lane 6 vs. lane 3 and Figure 4H; lane 6 vs. lane 2). On the contrary, USP11 depletion increased the polyubiquitination of the mutant SFTPC*^I73T^* protein (Figure 4G; lane 7 and Figure 4H; lane 8). The catalytically inactive USP11 mutant had no effect on the ubiquitination status of either the SFTPC or SFTPC*^I73T^* proteins (Figure 4H; lane 3 and lane 7). Additionally, USP11 was observed to have similar deubiquitinating activity on the ubiquitination status of the ectopically expressed Myc-SFTPC*^I73T^* mutant protein (Figure S7B).

Next, we estimated the half-life of the mutant SFTPC*^I73T^* protein to assess its protein turnover. Those results demonstrated that the half-life of SFTPC*^I73T^* was significantly longer than that of the SFTPC protein (Figure S8). Moreover, USP11 overexpression further prolonged the half-life of the SFTPC*^I73T^* protein (Figure 4I; lanes 5-8), whereas the USP11 catalytic mutant did not affect the half-life of the SFTPC*^I73T^* protein (Figure 4I; lanes 9-12). In contrast, the depletion of USP11 significantly shortens the half-life of the SFTPC*^I73T^* protein (Figure 4J; lanes 9-12), suggesting that the deubiquitinating activity of USP11 extends the half-life of the mutant SFTPC*^I73T^* protein, resulting in the accumulation of misfolded protein through retarded protein turnover.

### USP11 enhances the fibrogenic effects of the SFTPC*^I73T^* mutant in vitro under BLM-induced stress

**Figure 5:**
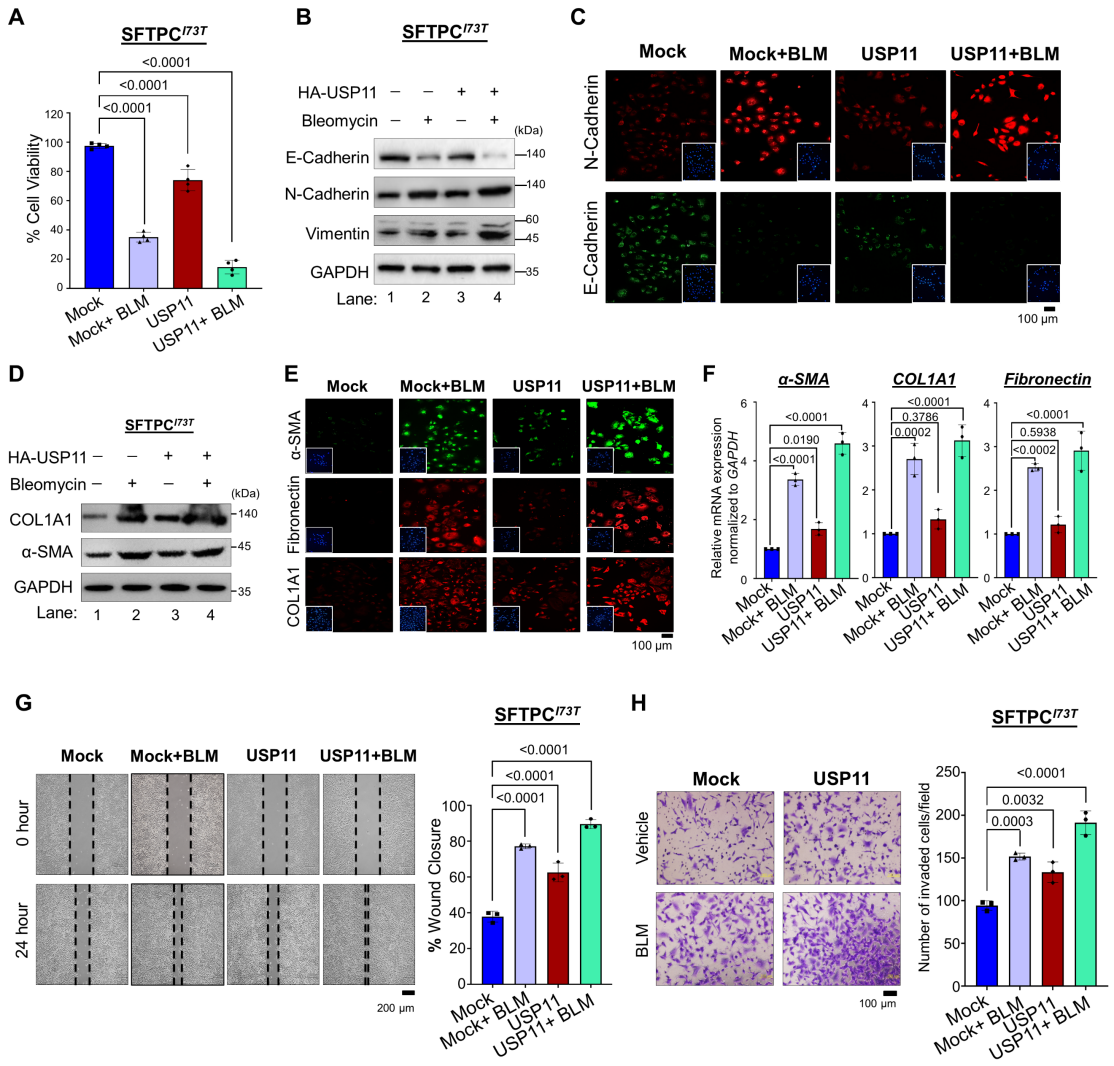
** The effect of USP11 on SFTPC*^I73T^*-mediated fibrosis under BLM-induced stress.** All the experiments were carried out in BEAS-2B-SFTPC*^I73T^* cells. **(A)** The cell viability analysis was conducted on SFTPC*^I73T^* cells treated with either BLM alone or a combination of BLM and USP11 transduction. Data are presented as the mean and standard deviation of four independent experiments (n = 4). **(B-F)** The experimental group was analyzed by **(B)** western blot and **(C)** immunofluorescence for EMT markers, Scale bar: 100 μm. The same samples were analyzed by **(D)** western blot, **(E)** immunofluorescence, Scale bar: 100 μm and **(F)** qRT-PCR for fibrosis markers. Data are presented as the mean and standard deviation of three independent experiments (n = 3). SFTPC*^I73T^* cells treated with either BLM alone or a combination of BLM and USP11 transduction were subjected to **(G)** migration, Scale bar: 200 μm and **(H)** invasion assays, Scale bar: 100 μm. Data are presented as the mean and standard deviation of three independent experiments (n = 3). Two-way ANOVA followed by Tukey's post hoc test was used with the indicated *P*-values. All the experiments from **(A-H)** used DMSO as a control reagent and an empty vector as the mock control, and USP11-transduced cells were treated with either DMSO (control) or BLM reagent.

To further corroborate our finding that USP11 exhibits stronger interaction, higher deubiquitinating activity, and longer extension of the half-life of SFTPC*^I73T^*, compared with the wild-type SFTPC protein, we delineate our study by focusing on the USP11-mediated regulation of the pathogenic function of the SFTPC*^I73T^* mutant during fibrogenesis. We carried out this investigation in BEAS-2B cells stably expressing the mutant SFTPC*^I73T^* protein in the presence and absence of USP11. We estimated the expression level of USP11 by treating the BEAS-2B cells with BLM, a chemical that induces PF. Interestingly, the BLM-treated cells showed a significant increase in USP11 expression compared with the untreated cells (Figure S9). Therefore, we stably overexpressed an empty vector (mock) or USP11 in BEAS-2B-SFTPC^I73T^ cells (Figure S10), treated them with either DMSO (control) or BLM, and then conducted several experiments to investigate the effects of USP11 on the pathological behavior of the SFTPC*^I73T^* mutant. In fibrotic conditions, BLM treatment alone suppressed cell viability. Moreover, USP11 overexpression in conjunction with BLM treatment exacerbated cell death rates in BEAS-2B-SFTPC^I73T^ cells (Figure 5A).

We investigated regulation of the epithelial-to-mesenchymal transition (EMT) by the *SFTPC^I73T^* mutation in BLM-treated BEAS-2B-SFTPC^I73T^ cells in the presence and absence of USP11. Compared with cells treated with BLM alone, the combination of BLM and USP11 dramatically decreased the expression of E-cadherin (Figure 5B; lane 4 vs. lane 2). Similarly, combined BLM and USP11 treatment resulted in a higher expression of N-cadherin and vimentin than BLM treatment alone (Figure 5B; lane 4 vs. lane 2). Our results were well supported by an immunofluorescence assay showing significantly low expression of E-cadherin and high expression of N-cadherin in cells treated with both USP11 and BLM (Figure 5C). Furthermore, USP11 overexpression in BLM-treated cells resulted in a significant increase in the expression of fibrotic markers such as alpha-smooth muscle actin (α-SMA) and collagen type I (COL1A1) compared with cells treated with BLM alone (Figure 5D; Figure S11). The immunostaining of fibrotic markers was significantly greater in cells treated with both USP11 and BLM than in cells treated with BLM alone (Figure 5E). Additionally, the mRNA expression analysis of *α-SMA*, *COL1A1*, and *fibronectin* confirmed the upregulation of fibrotic markers in the USP11-BLM treatment group, compared with the controls (Figure 5F). Furthermore, cell migration and cell invasion were tested using wound healing and transwell invasion assays. The combination of USP11 overexpression and BLM treatment produced increased cell migration and invasion, compared with cells treated with BLM alone (Figure 5G and 5H). These data suggest that USP11 enhances the pathogenic effects of the SFTPC*^I73T^* mutation by promoting cellular EMT processes that contribute to fibrosis progression.

### Generation of hiPSCs carrying the *SFTPC^I73T^* mutation by CRISPR/Cas9

**Figure 6:**
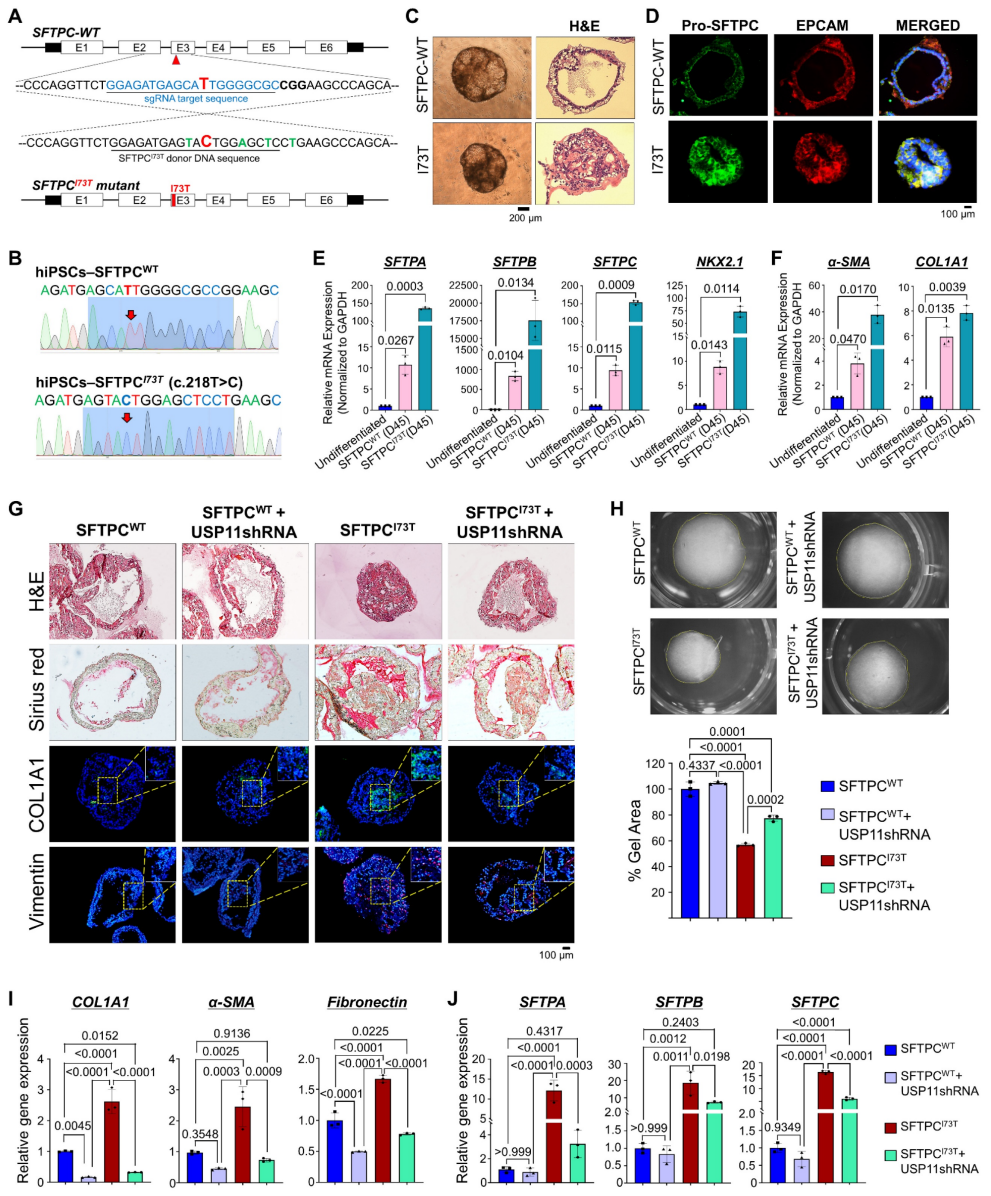
** The effect of USP11 depletion on SFTPC^I73T^-mediated epithelial cell abnormalities and fibrosis in alveolar organoids.** (A) Schematic representation of sgRNA targeting the *SFTPC* gene and the donor DNA design. The red arrowhead indicates the position of sgRNA1, designed to target the *SFTPC* gene. The blue font represents the sgRNA target sequence. A silent mutation represented in green font was introduced into the donor DNA to facilitate screening of the mutated clones by RFLP. The bold red font indicates the T to C missense mutation introduced by the HDR method. (B) Sanger sequencing analysis of the SFTPC*^I73T^* (T218C) missense mutations on the *SFTPC* gene. Bold letters indicate the T to C missense mutation introduced by the HDR method. (C-D) Characterization of AOs by (C) morphology by bright field and H&E staining, Scale bar: 200 μm (D) IHC staining using Pro-SFTPC and EPCAM, Scale bar: 100 μm (E-F) qRT-PCR analysis of AOs on day 45 for (E) the differentiation markers SFTPA, SFTPB, SFTPC and NKX2.1 and (F) the fibrosis markers α-SMA and COL1A1. Data are presented as the mean and standard deviation of three independent experiments (n = 3). (G-J) The effect of USP11 depletion on SFTPC^I73T^-mediated epithelial cell abnormalities and fibrosis in alveolar organoids was characterized by (G) H&E staining, Sirius red staining and immunostaining with COL1A1 and vimentin, Scale bar: 100 μm. (H) collagen gel contraction assay, scale bar 2 mm (I) qRT-PCR analysis of AOs using fibrosis markers *COL1A1*, *α-SMA*, and *Fibronectin* (J) qRT-PCR analysis of AOs using differentiation markers *SFTPA*, *SFTPB*, and *SFTPC*. Data are presented as the mean and standard deviation of three independent experiments (n = 3). Two-way ANOVA followed by Tukey's post hoc test was used with the indicated *P*-values.

To gain further insights into the regulatory role of USP11 in the SFTPC*^I73T^* mutation-associated pathogenesis of human PF, we used CRISPR/Cas9-mediated genome editing to generate a hiPSCs line carrying the homozygous I73T mutation using double-stranded donor DNA. The donor DNA was designed with four silent mutations and the I73T point mutation, which added a *ScaI* restriction enzyme site to facilitate screening for the mutant cell line (Figure 6A). Next, we designed three individual sgRNAs that specifically targeted the *SFTPC* locus adjacent to the I73T mutation site and tested their DNA cleavage efficiency using the T7E1 assay. The sgRNA1 demonstrated higher DNA cleavage efficiency than the other sgRNAs (Figure S12A). Therefore, sgRNA1 was selected and used, along with Cas9-GFP and donor DNA, for the hiPSCs transfection. After transfection, GFP-expressing hiPSCs were sorted using flow cytometry and seeded onto 96-well plates for cell expansion, followed by screening through the RFLP method (Figure S12B). The HDR-directed single cell-derived knock-in of the I73T mutant clone was screened by *ScaI* restriction endonuclease digestion (Figure S12C). Sanger sequencing of the edited hiPSCs showed the expected A to T substitution in exon 2 of the *SFTPC* gene, confirming the introduction of the I73T mutation on both alleles (hereafter hiPSCs-SFTPC^I73T/I73T^) (Figure 6B). Alkaline phosphatase staining (Figure S13A), western blot analyses (Figure S13B), immunostaining (Figure S13C), and qRT-PCR analyses (Figure S13D) for key pluripotency markers revealed no significant differences in the expression levels of these markers in the hiPSCs-SFTPC^I73T/I73T^ and wild-type hiPSCs, suggesting that the CRISPR/Cas9-mediated genome editing did not affect the pluripotency status of the hiPSCs-SFTPC^I73T/I73T^.

### hiPSCs-*SFTPC^I73T/I73T^*-derived AOs for modeling pulmonary fibrosis

Next, we wished to develop in vitro human AOs carrying the SFTPC*^I73T^* mutation to characterize its pathological behavior during PF. To this end, we differentiated the hiPSCs-SFTPC^I73T/I73T^ cells into AECs to generate AOs containing hiPSCs-derived SFTPC*^I73T^*. We performed the stepwise differentiation of hiPSCs into AOs using an optimized protocol that was previously described 32-34. We obtained effective differentiation of the alveolar epithelium from the hiPSCs-SFTPC^I73T/I73T^ cells, with functional AECs. After being placed in AEC maturation medium, the aggregates were cultured for nine more days to develop into AOs and then sampled on day 30 for analysis. The resulting AOs showed multiple alveoli and layers of epithelial cells resembling an alveolar sac, and they expressed differentiation markers (Figure 6C and 6D).

The hiPSCs-SFTPC^WT^-derived AOs (hereafter hiPSCs-SFTPC^WT^-AOs) exhibited a monolayer epithelial sphere morphology, whereas the hiPSCs-SFTPC^I73T^-derived AOs (hereafter hiPSCs-SFTPC^I73T^-AOs) lacked lumens and displayed disorganized and aberrant alveolar morphology, as shown by H&E staining (Figure 6C). Additionally, in line with a previous report 10, immunostaining revealed normal cytoplasmic localization of pro-SFTPC in the hiPSCs-SFTPC^WT^-AOs (Figure 6D; upper panel), whereas the hiPSCs-SFTPC^I73T^-AOs showed mislocalization of pro-SFTPC in the plasma membrane (Figure 6D; lower panel), suggesting that the hiPSCs-SFTPC^I73T^-AOs successfully expressed the mislocalized mutant SFTPC*^I73T^* protein 10, 35. The mRNA expression analysis showed that the AT2 markers (SFTPA, SFTPB, SFTPC) and alveolar epithelial progenitor marker (NKX2.1) were elevated in the hiPSCs-SFTPC^I73T^-AOs (Figure 6E). Moreover, the expression of fibrotic markers (α-SMA and COL1A1) was also elevated in the hiPSCs-SFTPC^I73T^-AOs, compared with the hiPSCs-SFTPC^WT^-AOs (Figure 6F), suggesting AT2 abnormalities in the hiPSCs-SFTPC^I73T^-AOs. Overall, these data demonstrate that we successfully generated hiPSCs-SFTPC^I73T^-AOs for modelling PF showing the altered AT2 phenotype associated with the SFTPC*^I73T^* mutation.

### Depletion of USP11 ameliorates SFTPC^I73T^-mediated epithelial cell abnormalities and fibrosis in alveolar organoids

Next, we wanted to examine the regulatory role of the USP11-mediated stabilization of the SFTPC*^I73T^* mutant and its impact on the development of PF. Therefore, we depleted USP11 from both hiPSCs-SFTPC^WT^ and hiPSCs-SFTPC^I73T^ cells (Figure S14). These cells were differentiated into AT2-derived AOs to assess fibrotic changes at both the transcript and protein levels. The histological analysis using H&E staining showed that the reduced lumen area in the hiPSCs-SFTPC^I73T^-AOs was restored upon USP11 depletion (Figure 6G; upper panel). Additionally, sirius red staining of the hiPSCs-SFTPC^I73T^-AOs revealed substantial collagen deposition, compared with hiPSCs-SFTPC^WT^-AOs (Figure 6G; middle panel). Notably, USP11 depletion produced a marked reduction in Sirius red intensity in the hiPSCs-SFTPC^I73T^-AOs (Figure 6G; middle panel), suggesting that the knockdown of USP11 decreases collagen deposition. Additionally, the immunostaining analysis showed increased expression of fibrotic marker COL1A1 and EMT marker vimentin in the hiPSCs-SFTPC^I73T^-AOs (Figure 6G), while the USP11 depletion reduced those levels (Figure 6G).

One of the key phenotypes indicative of PF is the contraction of lung tissue, driven by the contraction of activated fibroblasts 36. To investigate the effects of the SFTPC*^I73T^* mutation on the AO phenotypes, we performed a collagen gel contraction assay that evaluated the contraction of the cultivation matrix dependent on the interaction between alveolar epithelial and mesenchymal cells. Notably, the presence of the SFTPC*^I73T^* mutation in AOs resulted in high collagen gel contraction compared with the wild-type SFTPC, and USP11 depletion reversed that phenotype (Figure 6H), indicating that USP11 depletion mitigates SFTPC*^I73T^*-mediated fibrosis. Furthermore, the mRNA expression analysis of hiPSCs-SFTPC^I73T^-AOs revealed elevated levels of fibrotic markers (COL1A1, α-SMA, and fibronectin) compared with the hiPSCs-SFTPC^WT^-AOs (Figure 6I). It is interesting to note that the USP11-depleted hiPSCs-SFTPC^I73T^-AOs displayed dramatically reduced expression of those genes, which had been elevated as a result of the SFTPC*^I73T^* mutation (Figure 6I). Similarly, the EMT markers N-cadherin and vimentin were upregulated, and E-cadherin was downregulated in the hiPSCs-SFTPC^I73T^-AOs; however, those changes were reversed by USP11 depletion (Figure S15).

The elevated levels of AT2 markers (SFTPA, SFTPB, and SFTPC) in SFTPC*^I73T^* AOs suggest dysregulated cellular function and cellular stress due to the mutation, which aligns with the development of PF 10. Therefore, we assessed the effect of USP11 depletion on the expression of those AT2 markers in hiPSCs-SFTPC^I73T^-AOs. Consistent with a previous report 10, our mRNA expression analysis demonstrated that the SFTPC*^I73T^* mutation led to a significant increase in AT2 markers in the hiPSCs-SFTPC^I73T^-AOs, compared with hiPSCs-SFTPC^WT^-AOs (Figure 6J). Notably, USP11 depletion in hiPSCs-SFTPC^I73T^-AOs decreased the expression of those AT2 markers (Figure 6J). Overall, these findings suggest that USP11 depletion might alleviate the AEC abnormalities associated with the SFTPC*^I73T^* mutation.

### Pharmacological inhibition of USP11 exhibits a therapeutic effect on TGF-β-induced PF in hiPSCs-SFTPC^I73T^-AOs

**Figure 7:**
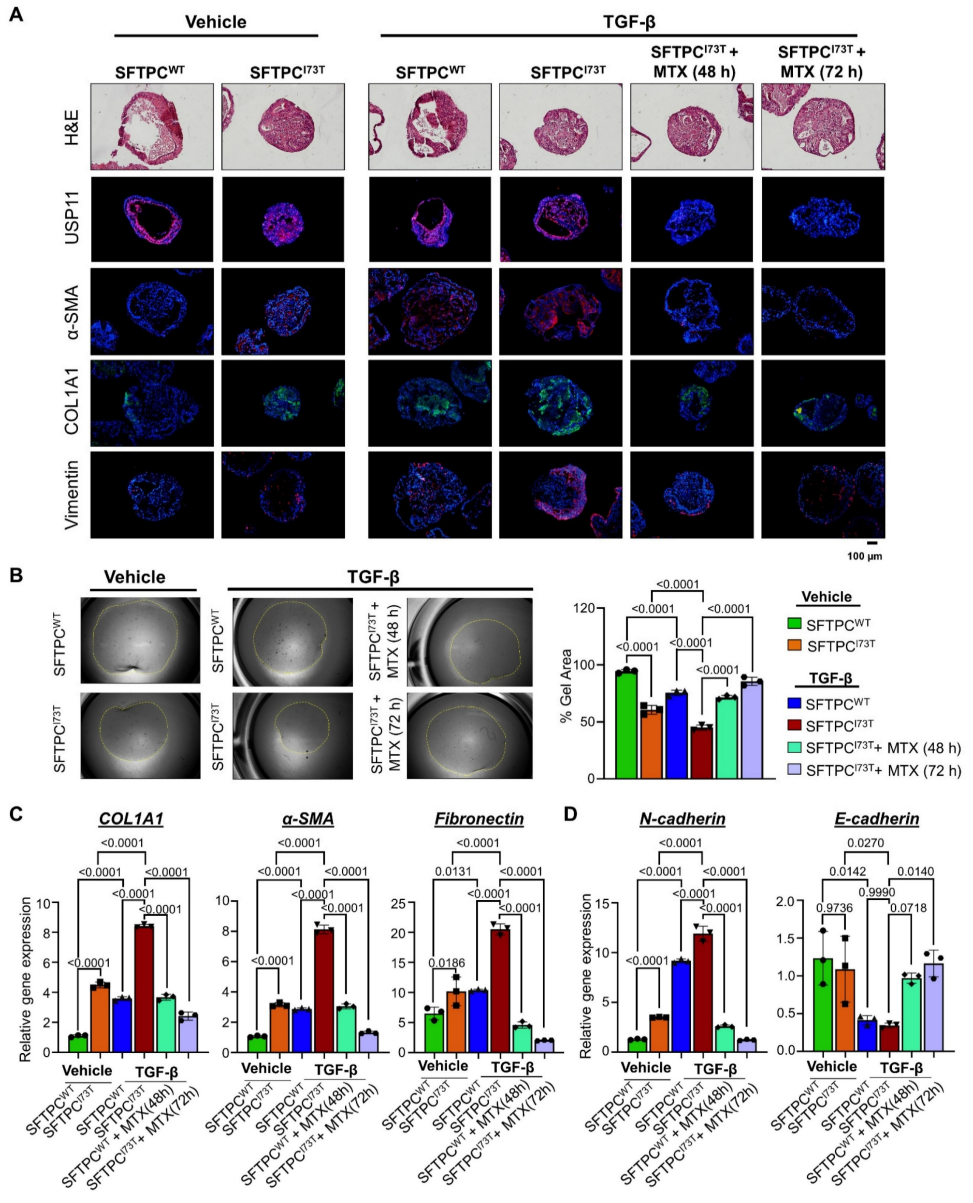
** Inhibition of USP11 mitigates TGF-β-induced PF in hiPSCs-SFTPC^I73T^-AOs.** The TGF-β (25 ng/mL) and MTX (6 µM) were co-treated on AOs and analyzed for fibrotic changes at the protein and transcript levels. **(A)** The effect of the USP11 inhibitor (MTX) on SFTPC^I73T^-mediated epithelial cell abnormalities and fibrosis in alveolar organoids was characterized by H&E staining and immunostaining with USP11, α-SMA, COL1A1, and vimentin. Scale bar: 100 μm. **(B)** Collagen gel contraction assay, scale bar 2 mm **(C)** qRT-PCR analysis of AOs using fibrosis markers *COL1A1*, *α-SMA*, and *Fibronectin* **(D)** qRT-PCR analysis of AOs using differentiation markers EMT markers N-cadherin and E-cadherin. Data are presented as the mean and standard deviation of three independent experiments (n = 3). Two-way ANOVA followed by Tukey's post hoc test was used with the indicated *P*-values.

Our research indicates that USP11 plays a role in fibrosis within AOs by stabilizing the SFTPC*^I73T^* protein. Thus, we evaluated the effect of MTX, a clinically approved USP11 inhibitor 37, to evaluate the plausibility of targeting USP11 for the treatment of TGF-β-induced fibrosis in AOs. TGF-β demonstrated superior efficacy in inducing fibrotic markers in AOs compared to BLM (Figure S16). Thus, we co-treated TGF-β (25 ng/mL) and MTX (6 µM) for 48 h and 72 h and analyzed for fibrotic changes at both the protein and transcript level. The immunostaining analysis showed an increased expression of fibrotic markers α-SMA and COL1A1 along with the EMT marker vimentin in the TGF-β treated hiPSCs-SFTPC^I73T^-AOs (Figure 7A). Notably, TGF-β and MTX co-treated hiPSCs-SFTPC^I73T^-AOs exhibited a marked reduction in the expression of USP11 along with fibrotic markers (Figure 7A). Additionally, hiPSCs-SFTPC^I73T^-AOs treated with TGF-β had a higher collagen gel contraction compared to the wild-type SFTPC, while blocking USP11 with MTX reversed that phenotype (Figure 7B), indicating that USP11 inhibition lowers SFTPC*^I73T^*-driven pulmonary fibrosis. Furthermore, the mRNA expression analysis of TGF-β treated hiPSCs-SFTPC^I73T^-AOs revealed elevated levels of fibrotic markers (COL1A1, α-SMA, and fibronectin) (Figure 7C) and EMT markers (N-cadherin and E-cadherin) (Figure 7D) compared with the hiPSCs-SFTPC^WT^-AOs. In contrast, inhibition of USP11 in TGF-β treated hiPSCs-SFTPC^I73T^-AOs displayed a significant reduction in the expression of fibrotic and EMT marker genes (Figures 7C and 7D). Thus, our results demonstrate that MTX treatment reduces the fibrosis caused by TGF-β in hiPSCs-SFTPC^I73T^-AOs by targeting USP11.

### Pharmacological inhibition of USP11 exhibits therapeutic effect on BLM-induced PF in vivo

**Figure 8:**
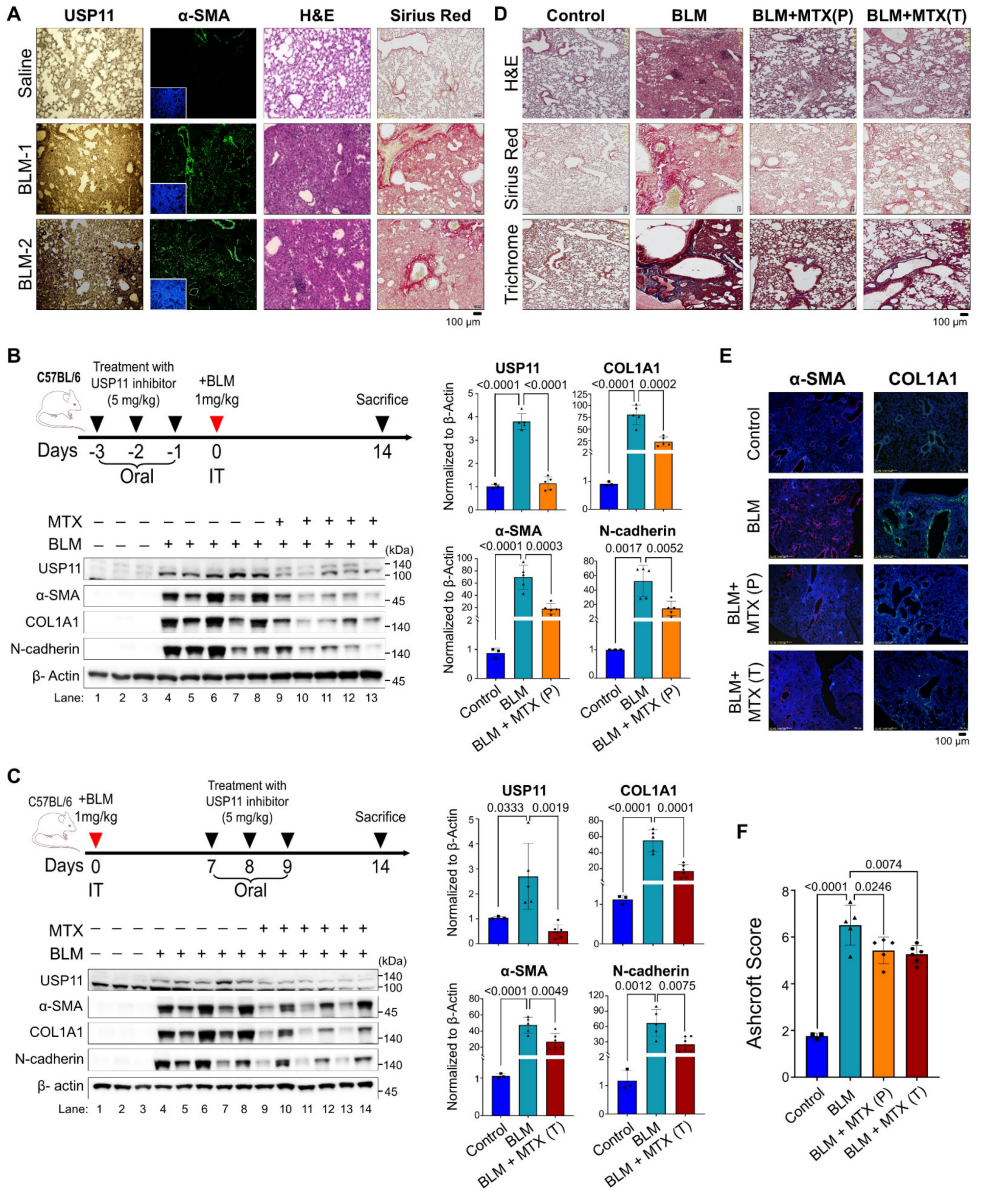
**Pharmacological inhibition of USP11 prevents and cures pulmonary fibrosis in BLM-induced mice model. (A)** The expression of USP11 and α-SMA were validated in BLM-induced mouse lung tissues by H&E and Sirius red staining. Scale bar: 100 μm. **(B)** C57BL/6 mice were treated with MTX orally for 3 days prior to BLM treatment. **(C)** C57BL/6 mice were treated with MTX orally for 3 days after BLM treatment. On day 14, the mice were sacrificed, and their lung tissues were subjected to western blot using the indicated antibodies. The protein band intensities for USP11, COL1A1, α-SMA, and N-cadherin were estimated using ImageJ software, normalized to β-actin, and represented graphically. **(D-F)** Lung tissues from the BLM-induced mice in both the preventive group (P) and therapeutic group (T) were sectioned and paraffin embedded. **(D)** H&E, Sirius red, and trichrome staining was performed, along with **(E)** immunohistochemical staining using α-SMA and COL1A1 antibodies. Scale bar: 100 μm. **(F)** Ashcroft score of paraffined lung tissue sections. All IHC images from mice lung tissues obtained from different experimental group were quantified with the Ashcroft score. Data are presented as the mean and standard deviation. Two-way ANOVA followed by Tukey's post hoc test was used with the indicated P values.

Next, we evaluated the pathological relevance of USP11 in vivo by conducting experiments with BLM-induced PF in mice (dose 1mg/kg) 38.The immunostaining analysis showed a significant increase in α-SMA in lung tissue from BLM-treated mice, indicating a fibrotic response (Figure 8A; left panel). Concurrently, a histopathological examination using H&E staining and Sirius red staining showed BLM-induced pathological changes and collagen deposition in the mouse lung tissues (Figure 8A). Interestingly, increased expression of Usp11 in lung tissues was observed following BLM treatment (Figure 8A). As a functional consequence of the BLM-induced upregulation of Usp11 expression in lung tissue, we speculated that the pharmacological inhibition of Usp11 might block PF progression. Therefore, we used MTX, a clinically approved and proven USP11 inhibitor 38, to determine the feasibility of targeting USP11 to prevent or treat BLM-induced lung fibrosis in mice.

To test the preventive approach, MTX (5mg/kg) was administered orally for 3 days prior to BLM treatment (Figure 8B), and to test the therapeutic strategy by blocking early intervention of fibrogenesis, MTX was administered on days 7, 8, and 9 post-BLM treatment (Figure 8C). On day 14, all the mice were sacrificed, and their lung tissues were analyzed. The results show that BLM treatment produced a significant decrease in the body weights of the mice, compared with the control group. Administration of MTX to the BLM-treated mice resulted in a smaller reduction in body weight than found in the BLM group, although the difference was not statistically significant (Figure S17A). Moreover, MTX administration improved the survival rate of mice treated with BLM (Figure S17B). Next, western blot analyses of the mouse lung tissues demonstrated that BLM treatment elevated USP11 and increased the levels of the fibrosis markers α-SMA, Col1A1, and N-cadherin (Figure 8B; lanes 4-8; Figure 8C; lanes 4-8). On the contrary, MTX treatment blocked USP11 expression and reduced the expression of fibrosis markers in both the preventive (Figure 8B; lanes 9-13) and therapeutic approaches (Figure 8C; lanes 9-14). Those results were further substantiated by the histopathological examination, wherein H&E staining showed the reversal of fibrosis morphology in MTX-treated mice from both the preventive and therapeutic groups, compared with BLM treated group (Figure 8D; top panel). Additionally, Sirius red and trichrome staining showed reduced collagen deposition in BLM/MTX-treated lungs (Figure 8D; middle and bottom panel). To support those findings, immunofluorescence staining of the mouse lung tissues revealed that treatment with BLM/MTX resulted in decreased expression of the fibrosis markers α-SMA and Col1A1 (Figure 8E) along with Usp11 (Figure S18) compared with BLM treatment alone. Additionally, the Ashcroft score was low in mouse lung tissues treated with BLM/MTX when compared with BLM treatment alone (Figure 8F). Collectively these results demonstrate that MTX treatment, administered preventively or therapeutically, alleviates BLM-induced lung fibrosis by targeting USP11.

## Discussion

More than 60 mutations have been identified in the AT2 cell-restricted gene *SFTPC*; those mutations are heterozygous and affect the biophysical activity of pulmonary surfactant 39. Among them, I73T is the most common pathogenic mutation, causing misprocessing of the pro-SFTPC protein and mislocalization to the plasma membrane as a partially cleaved intermediate. Similarly, the G100S mutation on *SFTPC*, which substitutes glycine for serine at position 100, causes cell death and leads to PF 40. The L118Q mutation in SFTPC results in PF by disrupting the interactions between SFTPC and other surfactant components, leading to pro-SFTPC misfolding and trapping in the endoplasmic reticulum without being transported to distal secretory compartments 41. The c.460+1 G → A mutation, which is thought to be the first human SFTPC mutation, produced an aberrant pro-protein that disturbed the metabolism of surfactant protein C 42, 43. The L184Q and C121G mutations are linked to severe disease phenotypes and impair the function of SFTPC 44, 45. Therefore, identifying the stabilizers of mutant SFTPC protein is a top priority to prevent the accumulation of misprocessed SFTPC proteins. In this study, we focused on pathogenic SFTPC*^I73T^* protein stabilization regulated through the ubiquitin proteasomal pathway and explored the therapeutic value of a unique DUB to counteract PF progression.

A growing body of evidence indicates that DUBs are associated with PF. For instance, USP13 acts as a protective factor in age-related PF, with the age-mediated depletion of USP13 promoting lung fibrosis through Beclin 1 deubiquitination 46. USP18 negatively regulates the ISGylation of FOXO3a, a protein that suppresses TGFβ1-induced fibronectin expression in human lung fibroblasts, suggesting that USP18 could be a potential target for treating IPF 47. In this study, we initiated our research by performing genome-wide screening for DUBs that regulate SFTPC protein levels. Compared with techniques using RNA interference (RNAi) or shRNA molecules, our screening technique is more robust and reliable because the CRISPR-Cas9 system causes permanent gene disruption. RNAi- and shRNA-based methods, on the other hand, produce transient partial repression that reverses after a few days. Our screening results identified USP11 and USP19 as DUBs that regulate SFTPC protein levels. The depletion of USP11 reduced SFTPC, while depletion of USP19 showed the opposite result by increasing SFTPC protein levels. This study focused on USP11 by suppressing its expression to assess its function in reducing the accumulation of the misfolded pathogenic mutant SFTPC protein, which is prominently observed in PF. It has been reported that USP11 stabilizes the GREM1 protein, a profibrotic mediator in IPF, so a potential link between USP11 and the progression of IPF had already been suggested 48-50. Furthermore, compared with normal lung tissues, lung tissues from IPF patients had higher expression of USP11 49. These observations motivated us to investigate the role of USP11 in PF. Here, we demonstrated that USP11 deubiquitinates and accumulates substantial quantities of mutant SFTPC^I73T^ protein in cells by extending its half-life (Figure 4). Given that the accumulation of the mutant SFTPC^I73T^ protein in ILD is linked to its prolonged half-life, a strategy to promote the rapid degradation of the mutant SFTPC^I73T^ protein may enhance therapeutic efficacy. This study demonstrated that the depletion of USP11 markedly decreases the half-life of the mutant SFTPC^I73T^ protein by promoting its protein degradation.

The ability of polarized epithelial cells to undergo molecular changes that give them the ability to produce extracellular matrix, including collagen and fibronectin, via the EMT, has been implicated in fibrosis and is associated with increased invasion and migration ability 51. It was reported that the L188Q *SFTPC* mutation is associated with EMT regulation because the expression of *SFTPC*-L188Q in A549 cells increased vimentin and N-cadherin expression and decreased E-cadherin expression 52. The same mutation increased the mesenchymal marker α-SMA, which is a hallmark of fibrosis formation. Using BLM treatment, we demonstrated that USP11 enhances SFTPC*^I73T^*-induced fibrosis and EMT processes by increasing the migration and invasion ability of BEAS-2B cells, suggesting that USP11 enhances the pathogenicity of the SFTPC*^I73T^* mutation.

Heterozygous expression of the mutant *SFTPC^I73T^* allele was reported in several cohorts of IPF and childhood interstitial lung disease patients 35, 53-55. However, Nureki et al. developed both heterozygous (*SFTPC^I73T/WT^*) and homozygous (*SFTPC-^I73T/I73T^*) mouse embryos and compared the pathological behavior associated with both 17. Lung tissues from the *SFTPC^I73T^* alleles exhibited arrested lung morphogenesis in late sacculation, and the extent of lung disruption was higher in homozygous embryos than in heterozygous embryos. Furthermore, homozygous embryos had a significantly lower expression of aquaporin 5 and a significantly higher expression of pro SFTPC*^I73T^* protein, indicating a higher degree of disruption in AT2/AT1 homeostasis 17. Therefore, we introduced a homozygous SFTPC*^I73T^* mutation into hiPSCs using the CRISPR/Cas9 system to obtain increased, allele-dependent expression of the SFTPC protein.

Preclinical modelling of PF is challenging due to a lack of models that can recapitulate the features of human alveolar tissue and the pathophysiology of the disease. Currently, 3D AOs and spheroids are models that resemble pulmonary AECs and are used to investigate the onset of lung fibrosis. Our development of in vitro human AOs carrying the SFTPC*^I73T^* mutation thus holds great promise for elucidating the role of USP11 in SFTPC*^I73T^*-induced fibrosis. The hiPSCs-derived AOs carrying the SFTPC*^I73T^* mutation showed disorganized and aberrant alveolar morphology, with a restricted lumen and mislocalization of the mutant SFTPC protein to the plasma membrane. Furthermore, in line with a previous report 10, AOs carrying the SFTPC*^I73T^* mutation showed increased expression of AT2 markers, indicating an aberrant response of alveolar epithelial cells caused by the mutation. The SFTPC*^I73T^* mutation results in impaired protein folding and accumulation, leading to cellular stress and impaired surfactant function that in turn contributes to alveolar instability and damage 43, 56. This dysfunction is associated with elevated AT2 markers and can lead to fibrotic changes, such as increased expression of fibrosis markers and collagen deposition 57-60. Similarly, we observed increased collagen deposition and elevated fibrosis markers in the hiPSCs-SFTPC*^I73T^*-AOs. However, USP11 depletion counteracted the pathological effects of the I73T mutation and restored normal AT2 function. Furthermore, pharmacological inhibition of USP11 using MTX, a specific inhibitor of USP11, in AOs significantly reduced fibrotic markers both at the protein and mRNA level. Finally, inhibition of USP11 using MTX in mice significantly reduced fibrosis in their lung tissues. Future research in ILD primarily focuses on utilizing specific mutant* SFTPC* gene mouse models to examine the impact of USP11 inhibitors, thereby enhancing comprehension of the disease's genetic basis and facilitating the development of targeted therapies. In conclusion, our findings suggest that USP11 stabilizes and prevents SFTPC*^I73T^* protein degradation and eventually enhances its pathogenic outcomes during PF. Therefore, targeting USP11 could be a promising therapeutic strategy for mitigating the pathogenic effects of SFTPC mutations associated with the onset of PF.

## Materials and Methods

### Cell culture

HEK293 and BEAS-2B cells were maintained in DMEM (Gibco BRL, Rockville, MD, USA) supplemented with 10% fetal bovine serum (Gibco) and 1% penicillin and streptomycin (Gibco) at 37 °C in a humidified atmosphere with 5% CO2. The cells were passaged every 3-4 days, depending on cell confluence, using 0.25% Trypsin-EDTA (Gibco). hiPSCs (Cat. No. A18943PIS, GIBCO) were maintained on BD Matrigel (Cat. No. 356230, BD Biosciences)-coated dishes in StemFlex^TM^ medium (Cat. no. A3349401, Gibco) supplemented with 10 µM Y-27632 dihydrochloride (Cat. No. 1254, TOCRIS, Bristol, UK). The cell lines purchased from authorized repositories were authenticated by short, tandem, repeat profiling of the respective cell banks. Before being used in experiments, all cell lines were treated with Plasmocin mycoplasma eliminating reagent (Cat. No. ant-mpt1, InvivoGen) according to the manufacturer's instructions. Mycoplasma contamination was tested using a MycoAlert™ mycoplasma detection kit (Cat. No. LT07-118, Lonza Bioscience).

### Plasmids, sgRNAs, and shRNAs

The Myc-DDK-*SFTPC* plasmid was purchased from Origene (RC229661L3), and the DDK-tag was removed using site-directed mutagenesis by PCR (hereafter Myc-SFTPC). HA-tagged *USP11* (Addgene #46749), HA-tagged *USP11CS* (Addgene #46750), and pCAG-Cas9-GFP (Addgene #44719) plasmids were purchased from Addgene. The Myc- *SFTPC^I73T^* mutant was generated by using site-directed mutagenesis to replace isoleucine with threonine at position 73; the resulting vector was named Myc*-SFTPC^I73T^*. To screen DUBs with the potential to regulate SFTPC expression, a plasmid encoding Cas9-2a-mRFP-2a-PAC (N-acetyl-transferase puromycin resistance gene) and plasmids encoding sgRNAs were purchased from Toolgen (Seoul, South Korea). To generate sgRNAs targeting potential DUB candidates, the sequences were designed using a public tool (www.broadinstitute.org) and cloned into pRG2-CT sgRNA vectors 24, as described previously. Briefly, oligonucleotides with the *USP11* target sequence were synthesized (Bioneer, Seoul, South Korea), and T4 polynucleotide kinase was used to add terminal phosphates to the annealed oligonucleotides (Bio-Rad, CA, USA). The vector was digested using the *BsaI* restriction enzyme and ligated with the annealed oligonucleotides. The sgRNA-mediated USP11 depletion was implemented in immunoprecipitation and protein turnover assays.

For short hairpin RNA (shRNA) generation, lentiviral vector constructs and packaging plasmids were provided by Prof. Chung Hee Yong (Hanyang University, Seoul, South Korea). The shRNA-mediated USP11 depletion was implemented in TUBES assay and for lentiviral transduction of AOs. The target sequences for the sgRNAs targeting *USP11* and *SFTPC*, and the shRNAs used for *USP11* depletion are listed in Tables S1 and S2, respectively.

### Transfection and transduction

The cells were seeded and at 70-80% the confluence was transfected with the indicated constructs using polyethyleneimine (Polysciences, Inc. Cat no. 24765) and Lipofectamine 3000 (Cat no. L3000001, Thermo Fisher Scientific) or Lipofectamine 2000 (Cat no. 11668019, Life Technologies), according to the manufacturers' instructions. The cells were harvested 36-48 h post-transfection for further analysis.

Lentivirus particles were generated by co-transfecting HEK293 cells with the constructs (Control shRNA, USP11 shRNAs, HA-USP11, Myc-SFTPC-WT, or Myc- SFTPC***^I73T^***) and lentivirus packaging plasmids (pLP1, pLP2, and pLP-VSVG) in a 4:1:1:1 ratio. The supernatants were harvested 48 h post-transfection and either used to infect cells or stored at -80 °C. The supernatants were used to transduce cells at low confluence for 24 h in the presence of 10 ng/mL polybrene (Cat. No. TR-1003, Sigma Aldrich). The 48 h post-infection, the cells were selected using puromycin for two days to obtain cells expressing HA-USP11, shRNA targeting USP11, Myc-SFTPC, or Myc-SFTPC*^I73T^*.

### Antibodies and reagents

Mouse monoclonal antibodies against Flag (Anti-DDDDK-tag mAb, M185-3L, 1:1,000) were purchased from MBL Life Science; those against c-Myc (SC-40, 1:1,000), ubiquitin (sc-8017, 1:1,000), HA (sc-7392, 1:1,000), GAPDH (sc-32233, 1:1,000), USP11 (sc-365528, 1:1,000), COL1A1 (sc-293182, 1:500), vimentin (Abcam ab8978, 1:700), N-cadherin (Santa Cruz sc-8424, 1:1000), E-cadherin (Santa Cruz sc-21791, 1:1000), alpha-smooth muscle actin (α-SMA; Santa Cruz sc-53015, 1:500), ACTIN (Santa Cruz sc-47778, 1:5,000), and normal mouse IgG (sc-2025, 1:1,000) were purchased from Santa Cruz Biotechnology. Rabbit polyclonal antibodies against SFTPC (Cusabio CSB-PA021174GA01HU, 1:1000), SFTPC (Abcam ab90716, 1:500), USP19 (Proteintech 25768-1-AP, 1:1,000 ), Anti-Collagen 1 (Abcam ab21286, 1:500), α-SMA (Abcam ab124964, 1:500), OCT3/4 (ab18976; 1:500; Abcam Inc.), SOX2 (SC-365823, 1:250), SSEA4 (90231, 1:250; Millipore), fibronectin (CSB-PA13187C0Rb, 1:1,000), and 488/594-conjugated secondary antibodies (Cat. no. A21207 and Cat. no. A21203, 1:200; Life Technologies) were used.

Immunoprecipitation (IP) lysis buffer (Cat. no. 87787; Thermo Fisher), cell lysis buffer (Cat. no. R2002, Biosesang), protein 5X sample buffer (Cat. no. EBA-1052, Elpis Biotech), Protein A/G Plus agarose beads (sc-2003, Santa Cruz Biotechnology), protease inhibitor cocktail (Cat. no. 11836153001, Roche), the protein translation inhibitor cycloheximide (CHX; Cat. no. 239765, Merck), the proteasomal inhibitor MG132 (Cat. no. S2619, Selleckchem), the ubiquitin activating enzyme inhibitor MLN7243 (also called TAK243, Cat no. HY-100,487, Med Chem Express), puromycin (Cat. no. 12122530, Gibco), the USP11 inhibitor mitoxantrone dihydrochloride (MTX; Sigma-Aldrich, Cat no. M6545-10mg), the DUB inhibitor PR-619 (ab144641, Abcam), CCK-8 assay reagent (Dojindo Molecular Technologies, MD, USA), and 4', 6-diamidino-2-phenylindole (DAPI; Cat. no. H-1200, Vector Laboratories) were purchased and used.

### Immunoprecipitation

Cells were transfected with the indicated DNA constructs. At 36-48 h post-transfection, the cells were lysed in IP lysis buffer (25 mM Tris-HCl (pH 7.4), 150 mM sodium chloride, 1 mM EDTA, 1% NP-40, 5% glycerol, 1 mM PMSF, and protease inhibitor cocktail) for 20 min, and then the amount of protein was estimated using Bradford reagent. Cell lysate (2-3 mg) was immunoprecipitated using the indicated antibodies at 4 °C overnight. The following day, we incubated the lysates with 35 μL of protein agarose beads at 4 °C for 2 h. The agarose beads were washed with lysis buffer and eluted in 2X SDS sample loading buffer (5X SDS sample loading buffer containing 4% SDS, 20% glycerol, 10% 2-mercaptoethanol, 0.004% bromophenol blue, and 0.125 M Tris-HCl [pH 6]). The eluted samples were boiled at 95-100 °C for 5 min, separated on SDS-PAGE gels by western blotting, and analyzed using a ChemiDoc imaging system. Mouse IgG (ab-99697, 1: 10,000; Abcam) and rabbit IgG (CST-58802S, 1: 10,000; Cell Signaling Technology) light chain-specific secondary antibodies were used to prevent interference from heavy and light immunoglobulin chains in the binding assay.

### Tandem ubiquitin-binding entities assay

The ubiquitination status of SFTPC protein was determined using a tandem ubiquitin binding entities (TUBEs) assay (Cat. no. UM402, LifeSensors, PA, USA) as previously described. The HA-USP11 and USP11 shRNA transduced cells were lysed in IP lysis buffer containing 150 mM sodium chloride, 1% Triton X-100, 25 mM Tris (pH 7.5), 1 mM EDTA, 10% glycerol, and protease inhibitor cocktail. The lysed protein extracts were incubated with 20 µL of ubiquitin affinity matrices-TUBE2 at 4 °C for 3 h with rotation. The beads were then washed with IP lysis buffer, and samples were eluted in 30 µL of 2X SDS sample loading buffer (5X SDS sample loading buffer containing 4% SDS, 20% glycerol, 10% 2-mercaptoethanol, 0.004% bromophenol blue, 0.125 M Tris-HCl (pH 6.8)). The eluted samples were boiled at 95 °C-100 °C for 5 min, separated by SDS-PAGE, and analyzed by western blotting.

### Deubiquitination assay

Cells were transfected with HA-USP11, HA-USP11CS and sgRNA targeting USP11 constructs. The DUB activity of USP11 against endogenous and exogenous SFTPC protein was determined in BEAS-2B and HEK293 cells, respectively. The cells were treated with MG132 (10 µM/mL for 6 h) 48 h post-transfection and harvested. The cells were lysed for 20 min in a denaturing lysis buffer containing 150 mM sodium chloride, 1% Triton X-100, 1% sodium deoxycholate, 1% SDS, 50 mM Tris-HCl (pH 7.4), 2 mM EDTA, 1 mM PMSF, and protease inhibitor cocktail. Cell lysates (2-3 mg) were immunoprecipitated with the respective antibodies at 4 °C overnight and incubated with 35 μL of protein agarose beads for 2-3 h at 4 °C. The agarose beads were washed with lysis buffer, and the samples were eluted in 2X SDS sample loading buffer (5X SDS sample loading buffer containing 4% SDS, 20% glycerol, 10% 2-mercaptoethanol, 0.004% bromophenol blue, and 0.125 M Tris-HCl [pH 6]). The eluted samples were boiled at 95 °C-100 °C for 5 min, separated on SDS-PAGE gels, and analyzed by western blotting. To prevent non-specific binding of polyubiquitin molecules to the SFTPC protein, the protein-bound agarose beads were washed with lysis buffer containing 300 mM NaCl.

### Duolink proximity ligation assay (PLA)

The interaction between USP11 and SFTPC was observed using a Duolink *in situ* PLA kit (Cat. no. DUO92101, Sigma Aldrich) according to the manufacturer's instructions. BEAS-2B cells were fixed in 4% paraformaldehyde (PFA) for 10 min at room temperature and then treated with a blocking solution. The cells were treated with primary antibodies targeting SFTPC and USP11 for 1 h at 37 °C, and then they were incubated with PLA probes for 1 h at 37 °C in a humidified chamber. After three washes, ligation ligase solution was added, and the cells were incubated for 30 min at 37 °C. The slides were incubated in an amplified polymerase solution for 100 min at 37 °C in the dark. Finally, the cells were stained with mounting medium containing DAPI. A Leica fluorescence microscope was used to capture the fluorescent images (Leica, DM 5000B; Leica CTR 5000; Wetzlar, Germany).

### T7 endonuclease 1 assay

Genomic DNA was isolated using DNeasy blood & tissue kits (Promega, Madison, WI, USA) according to the manufacturer's protocols. The region of DNA containing the sgRNA target site was amplified by PCR and denatured and annealed by stepwise heating to form heteroduplex DNA. The heteroduplex DNA was then treated with 5 units of T7E1 (New England Biolabs, MA, USA) for 15 to 20 min at 37 °C, followed by 2% agarose gel electrophoresis. The band intensity was quantified using ImageJ software, and mutation frequencies were calculated using the following equation: mutation frequency (%) = 100 × (1 - [1 - fraction cleaved]1/2), where the fraction cleaved was the total relative density of the cleavage bands divided by the sum of the relative density of the cleaved and uncut bands. The oligonucleotide sequences used for PCR amplification and the PCR-amplicon sizes of the *SFTPC* and *USP11* genes and expected cleavage size after the T7E1 assay are summarized in Tables S3 and S4, respectively.

### Migration and invasion assay

Wound healing and cell invasion assay was performed as described previously 28, 61, 62. Briefly, BEAS-2B I73T cells were seeded and grown to near 90% confluence. Scratches were made with a sterile pipette tip, and the wounded cell layer was washed with PBS to remove floating cells and incubated with media containing vehicle or BLM at 37 ^°^C with 5% CO_2_. The cell migration abilities were compared at 0 h and 24 h post-scratch using a light microscope and quantified using ImageJ software.

The transwell invasion assay was performed to analyze the invasion rate of cells. Transwell chambers (0.8 µm pore size) were coated with Matrigel for 1 h at 37 ^°^C according to manufacturer's instructions. BEAS-2B I73T cells were seeded at the density of 3.0 X 10^4^ cells per transwell in 500 µL of serum free media in the top chamber. The media containing vehicle or BLM were added to the bottom chambers and incubated for 24 h. Following incubation, cells were fixed with ice-cold 100% methanol followed by staining with crystal violet (Cat. No. 2601-4125). Cell invasion was analyzed, and images were produced using a Leica DM5000B microscope. The cells were counted in three random fields using imageJ and data are presented graphically.

### Generation of the *SFTPC^I73T^* mutation in hiPSCs using CRISPR/Cas9

The hiPSCs were co-transfected with a plasmid encoding pCAG-Cas9-GFP, sgRNA targeting *SFTPC* or scrambled-sgRNA (mock control), and donor DNA at a 1:2:4 ratio using Lipofectamine stem transfection reagent (STEM00001, Thermo Fisher Scientific, MA, USA), according to the manufacturer's instructions. Later, cells expressing GFP were sorted using flow cytometry. The sorted cells were seeded into 96-well plates at 25 cells/well and incubated in a CO_2_ incubator at 37 °C. After 15 days, the wells were microscopically evaluated, and the single cell-derived colonies were selected. The selected colonies were dissociated using gentle cell dissociation reagent (Cat. No. 100-0485, Stem Cell Technologies) and reseeded into 24-well cell culture plates. A small portion of the selected colonies was used to isolate genomic DNA and screened for the I73T mutation in the *SFTPC* locus by RFLP analysis using *ScaI* restriction enzyme. The oligonucleotide sequences used for PCR amplification and the PCR-amplicon sizes of the *SFTPC* gene and expected amplicon size after the *ScaI* digestion assay are summarized in Tables S5. The *SFTPC^I73T^* mutant single cell-derived clones were expanded and stored in a liquid nitrogen tank after Sanger sequencing.

### Alkaline phosphatase staining

Alkaline phosphatase staining (ALP) was performed according to the manufacturer's protocol. Briefly hiPSCs were cultured for 5 days and cells were washed with PBS and fixed in 4% PFA for 15 min and then the cells were stained using an ALP detection kit (Cat. no. 86R-1KT, Sigma-Aldrich). The staining solution was added, and the cells were incubated in the dark for 1 h. Post-incubation the cells were washed twice with TBST, and colonies were examined for the appearance of red pink/red coloration. The stained colonies were imaged using a microscope (IX71, Olympus, Tokyo, Japan).

### Stepwise differentiation of hiPSCs into AECs

Stepwise direct AEC differentiation was performed as previously described 63, 64. Briefly, undifferentiated hiPSCs were mechanically passed and then plated in dishes coated with Matrigel at a density of 8-10 colonies. After 4-5 days, AEC differentiation was initiated through exposure to stepwise induction media.

### Magnetic-activated cell sorting and the generation of AOs from hiPSCs

AOs were generated using a combination of previously reported protocols with minor modifications 63, 65, 66. Briefly, the AEC cultures were dissociated with 0.8 U/ml collagenase B (Roche) for 1 h in a 37 °C incubator on day 21 of AEC differentiation, followed by treatment with a cell dissociation buffer (Gibco) for 10 min in a 37 °C water bath. The single cell suspension was blended with 20 μL of anti-human CD326 IgG microbeads (EpCAM; Miltenyi Biotec, Bergisch Gladbach, Germany) and then incubated at 4°C for 30 min. After two washes, the labeled cells were applied to an LS column (Miltenyi Biotec, Bergisch Gladbach, Germany), and the magnetically labeled EPCAM-positive cells were eluted. Lastly, a flow cytometry analysis was conducted to determine the purity of the isolated AECs. The EpCAM-positive cells were seeded into 96-well round-bottom plates (Corning, 2.5× 10^4^ cells per well) containing AEC maturation medium. After distribution, 1:15 diluted Matrigel (50 μl per well) was added to each well to improve adhesion between cells, and the plates were incubated overnight at 37 °C in a humidified atmosphere with 5% CO_2_ to allow aggregation. After the overnight incubation, the aggregates were transferred to 6-well low-attachment plates (Corning) containing fresh AEC maturation medium and cultured for nine days to establish AOs. To induce pulmonary fibrosis, we used TGF-β at an optimized concentration of 25 ng/mL in AOs for 72 h. To inhibit USP11 expression, MTX at an optimized concentration of 6 μM was used on AOs for 72 h.

### Immunofluorescence staining

BEAS-2B cells were grown on glass coverslips and incubated at 37 °C in a humidified atmosphere with 5% CO_2_. The cells were washed with phosphate-buffered saline (PBS, Gibco), fixed for 15 min using 4% PFA (Biosesang), and permeabilized in PBS containing 0.1% Triton X for 5 min at room temperature. The cells were washed, blocked in 3% bovine serum albumin, and stained with the indicated primary antibodies overnight at 4 °C. The next day, the cells were washed with PBS and incubated with Alexa Fluor 488-conjugated secondary antibodies for 1 h. The nuclei were stained with DAPI, and the cells were mounted using VectaShield (Vector Laboratories, CA, USA). The cells were then visualized, and images were produced using a Leica fluorescence microscope (Leica, DM 5000B; Leica CTR 5000; Wetzlar, Germany).

### Quantitative real-time reverse transcription PCR (qRT-PCR)

The Trizol reagent (Favorgen, Kaohsiung, Taiwan) was used to isolate total RNA from BEAS-2B cells. The RNA concentration from cells were determined using NanoDrop (company) and extracted RNA was reverse transcribed into cDNA using a SuperScript III First-Strand Synthesis System (Life Technologies, USA) with an oligo-dT primer according to the manufacturer's protocol. The qRT-PCR was then carried out in triplicate using Fast SYBR Green I Master Mix (Life Technologies) and a Step One Plus Real-Time PCR System (Life Technologies) with cDNA as a template.

For AOs total RNA was extracted using the RNeasy Mini kit (Qiagen, Düsseldorf, Germany), and cDNA was synthesized using TOPscripTM RT DryMIX (Enzynomics, Daejeon, Korea). PCR amplification was performed using a Step One Plus real-time PCR system (Applied Biosystems, Warrington, UK) and TOPrealTM qPCR 2X PreMIX (Enzynomics). All mRNA expressions were normalized to an internal control, GAPDH. The expression levels of the target gene mRNAs were calculated by comparing them to the expression levels of GAPDH using the 2-ΔΔCt method. Primer sequences for humans are shown in Table S6.

### Collagen gel contraction assay

Collagen gel contraction assay was performed as described previously 67, 68. Briefly, hiPSCs-SFTPC^WT^-AOs, hiPSCs-SFTPC^I73T^-AOs, USP11-depleted hiPSCs-SFTPC^WT^/SFTPC^I73T^-AOs using USP11-shRNA were isolated and mixed at the density of 3 x 10^5^ cells/ml with 0.75 mg/ml of rat tail collagen and were allowed to polymerize for 15 min at 37 ^º^C. After incubation, the gel was gently detached from the plates and cultured in AEC maturation media for 72 h at 37 ^º^C with 5% CO_2_. The area of each gel was measured using ImageJ software.

### BLM-induced PF model

Male C57BL/6J mice (23-25 g, 10-12 weeks old) were purchased from Dooyeol Biotech (Seocho, Seoul, Korea), and all animal experiments were approved by the Institutional Animal Care and Use Committee of Kangwon National University (IACUC NO. KW-240326-7). The PF animal model was induced by a direct intratracheal injection of 1 mg/kg BLM (Chemical Industry, Tokyo, Japan), and control mice received saline (50 μL) intratracheally. MTX (Selleckchem, Houston, USA), a USP11 inhibitor, was administered orally at 5 mg/kg for 3 days before the induction of PF or 3 days after the induction of PF. The mice were sacrificed on day 14, and lung tissue was harvested for PF assessment. The body weights of the mice were monitored throughout the experimental period.

### Immunohistochemistry of paraffin sections

Formalin-fixed Paraffin-embedded (FFPE) hiPSCs-SFTPC^WT^-AOs, hiPSCs-SFTPC^I73T^-AOs, and USP11-depleted hiPSCs-SFTPC^WT^/SFTPC^I73T^-AOs using USP11-shRNA and lung tissue were deparaffinized using xylene and alcohol gradient and slides were blocked for endogenous peroxidase activity by treatment with DAKO Real Peroxidase Blocking Solution for 40 min at room temperature (RT). After being blocked with serum-free, liquid protein block (Agilent, X0909), the slides were incubated with primary antibodies overnight at 4 °C. The slides were washed in PBS with 0.1% Tween, and fluorescently conjugated secondary antibodies were applied for 1 h at RT in the dark. After washing, the slides were covered with FluoroshieldTM mounting medium containing DAPI and photographed using a fluorescence microscope (Olympus, Tokyo, Japan). Samples from at least three animals/AOs were used in each group, and data were obtained from 3-5 fields per sample.

### Histological examination

Paraffin sections of hiPSCs-SFTPC^WT^-AOs, hiPSCs-SFTPC^I73T^-AOs, and USP11-depleted hiPSCs-SFTPC^WT^/SFTPC^I73T^-Aos using USP11-shRNA and lung tissues were rehydrated sequentially through ethanol and exposed to hematoxylin and eosin (H&E) to counterstain the nuclei and cytoplasm. Then, they were mounted with Permount mounting medium (Fisher Scientific). Sirius red staining (Abcam, ab150681, UK) and Masson's trichrome (Empire Genomics, #BPK2916, Buffalo, NY, USA) staining were performed according to the manufacturers' instructions. For histological quantification, the Ashcroft score was used in a blinded fashion, with 0-1 indicating no fibrosis, 2-3 indicating minimal fibrosis, 4-5 indicating moderate fibrosis, and 6-8 indicating severe fibrosis.

### Statistical analysis

Statistical analyses and graphical presentation were performed using GraphPad Prism 9.0. All results are presented as the means and standard deviations of at least three independent experiments unless otherwise stated in the figure legends. Each error bar represents the standard deviations. Comparisons between two groups were analyzed using Student's t-test. Experiments involving three or more groups were analyzed by one-way or two-way analysis of variance (ANOVA) followed by Tukey's post hoc test. *P*-values <0.05 were regarded as statistically significant.

## Supplementary Material

Supplementary figures and tables.

## References

[1] Raghu G, Chen SY, Yeh WS, Maroni B, Li Q, Lee YC (2014). Idiopathic pulmonary fibrosis in US Medicare beneficiaries aged 65 years and older: incidence, prevalence, and survival, 2001-11. Lancet Respir Med.

[2] King TE Jr, Pardo A, Selman M (2011). Idiopathic pulmonary fibrosis. Lancet.

[3] Cottin V, Le Pavec J, Prévot G, Mal H, Humbert M, Simonneau G (2010). Pulmonary hypertension in patients with combined pulmonary fibrosis and emphysema syndrome. Eur Respir J.

[4] Flaherty KR, Travis WD, Colby TV, Toews GB, Kazerooni EA, Gross BH (2001). Histopathologic variability in usual and nonspecific interstitial pneumonias. Am J Respir Crit Care Med.

[5] Su W, Guo Y, Wang Q, Ma L, Zhang Q, Zhang Y (2024). YAP1 inhibits the senescence of alveolar epithelial cells by targeting Prdx3 to alleviate pulmonary fibrosis. Exp Mol Med.

[6] Günther A, Korfei M, Mahavadi P, von der Beck D, Ruppert C, Markart P (2012). Unravelling the progressive pathophysiology of idiopathic pulmonary fibrosis. Eur Respir Rev.

[7] Borok Z, Danto SI, Lubman RL, Cao Y, Williams MC, Crandall ED (1998). Modulation of t1alpha expression with alveolar epithelial cell phenotype in vitro. Am J Physiol.

[8] Foster CD, Varghese LS, Skalina RB, Gonzales LW, Guttentag SH (2007). In vitro transdifferentiation of human fetal type II cells toward a type I-like cell. Pediatr Res.

[9] Tomos IP, Tzouvelekis A, Aidinis V, Manali ED, Bouros E, Bouros D (2017). Extracellular matrix remodeling in idiopathic pulmonary fibrosis. It is the 'bed' that counts and not 'the sleepers'. Expert Rev Respir Med.

[10] Alysandratos KD, Russo SJ, Petcherski A, Taddeo EP, Acín-Pérez R, Villacorta-Martin C (2021). Patient-specific iPSCs carrying an SFTPC mutation reveal the intrinsic alveolar epithelial dysfunction at the inception of interstitial lung disease. Cell Rep.

[11] Cameron HS, Somaschini M, Carrera P, Hamvas A, Whitsett JA, Wert SE (2005). A common mutation in the surfactant protein C gene associated with lung disease. J Pediatr.

[12] Dickens JA, Rutherford EN, Abreu S, Chambers JE, Ellis MO, van Schadewijk A (2022). Novel insights into surfactant protein C trafficking revealed through the study of a pathogenic mutant. Eur Respir J.

[13] Beers MF, Kim CY, Dodia C, Fisher AB (1994). Localization, synthesis, and processing of surfactant protein SP-C in rat lung analyzed by epitope-specific antipeptide antibodies. J Biol Chem.

[14] Beers MF, Bates SR, Fisher AB (1992). Differential extraction for the rapid purification of bovine surfactant protein B. Am J Physiol.

[15] Brasch F, Ten Brinke A, Johnen G, Ochs M, Kapp N, Müller KM (2002). Involvement of cathepsin H in the processing of the hydrophobic surfactant-associated protein C in type II pneumocytes. Am J Respir Cell Mol Biol.

[16] Beers MF, Hawkins A, Maguire JA, Kotorashvili A, Zhao M, Newitt JL (2011). A nonaggregating surfactant protein C mutant is misdirected to early endosomes and disrupts phospholipid recycling. Traffic.

[17] Nureki SI, Tomer Y, Venosa A, Katzen J, Russo SJ, Jamil S (2018). Expression of mutant Sftpc in murine alveolar epithelia drives spontaneous lung fibrosis. J Clin Invest.

[18] Harrigan JA, Jacq X, Martin NM, Jackson SP (2018). Deubiquitylating enzymes and drug discovery: emerging opportunities. Nat Rev Drug Discov.

[19] Kitamura H (2023). Ubiquitin-Specific Proteases (USPs) and Metabolic Disorders. Int J Mol Sci.

[20] Chen R, Zhang H, Li L, Li J, Xie J, Weng J (2024). Roles of ubiquitin-specific proteases in inflammatory diseases. Front Immunol.

[21] Li S, Zhao J, Shang D, Kass DJ, Zhao Y (2018). Ubiquitination and deubiquitination emerge as players in idiopathic pulmonary fibrosis pathogenesis and treatment. JCI Insight.

[22] Yi XM, Li M, Chen YD, Shu HB, Li S (2022). Reciprocal regulation of IL-33 receptor-mediated inflammatory response and pulmonary fibrosis by TRAF6 and USP38. Proc Natl Acad Sci U S A.

[23] Wang W, Bale S, Wei J, Yalavarthi B, Bhattacharyya D, Yan JJ (2022). Fibroblast A20 governs fibrosis susceptibility and its repression by DREAM promotes fibrosis in multiple organs. Nat Commun.

[24] Das S, Chandrasekaran AP, Suresh B, Haq S, Kang JH, Lee SJ (2020). Genome-scale screening of deubiquitinase subfamily identifies USP3 as a stabilizer of Cdc25A regulating cell cycle in cancer. Cell Death Differ.

[25] Chandrasekaran AP, Kaushal K, Park CH, Kim KS, Ramakrishna S (2021). USP32 confers cancer cell resistance to YM155 via promoting ER-associated degradation of solute carrier protein SLC35F2. Theranostics.

[26] Haq S, Sarodaya N, Karapurkar JK, Suresh B, Jo JK, Singh V (2022). CYLD destabilizes NoxO1 protein by promoting ubiquitination and regulates prostate cancer progression. Cancer Lett.

[27] Baker BM, Chen CS (2012). Deconstructing the third dimension: how 3D culture microenvironments alter cellular cues. J Cell Sci.

[28] Karapurkar JK, Kim MS, Colaco JC, Suresh B, Sarodaya N, Kim DH (2023). CRISPR/Cas9-based genome-wide screening of the deubiquitinase subfamily identifies USP3 as a protein stabilizer of REST blocking neuronal differentiation and promotes neuroblastoma tumorigenesis. J Exp Clin Cancer Res: CR.

[29] Leuchowius KJ, Clausson CM, Grannas K, Erbilgin Y, Botling J, Zieba A (2013). Parallel visualization of multiple protein complexes in individual cells in tumor tissue. Mol Cell Proteomics.

[30] Jumper J, Evans R, Pritzel A, Green T, Figurnov M, Ronneberger O (2021). Highly accurate protein structure prediction with AlphaFold. Nature.

[31] Pettersen EF, Goddard TD, Huang CC, Meng EC, Couch GS, Croll TI (2021). UCSF ChimeraX: Structure visualization for researchers, educators, and developers. Protein Sci.

[32] Jacob A, Morley M, Hawkins F, McCauley KB, Jean JC, Heins H (2017). Differentiation of Human Pluripotent Stem Cells into Functional Lung Alveolar Epithelial Cells. Cell Stem Cell.

[33] Jacob A, Vedaie M, Roberts DA, Thomas DC, Villacorta-Martin C, Alysandratos KD (2019). Derivation of self-renewing lung alveolar epithelial type II cells from human pluripotent stem cells. Nat Protoc.

[34] Kim JH, An GH, Kim JY, Rasaei R, Kim WJ, Jin X (2021). Human pluripotent stem-cell-derived alveolar organoids for modeling pulmonary fibrosis and drug testing. Cell Death Discov.

[35] Brasch F, Griese M, Tredano M, Johnen G, Ochs M, Rieger C (2004). Interstitial lung disease in a baby with a de novo mutation in the SFTPC gene. Eur Respir J.

[36] Tomasek JJ, Gabbiani G, Hinz B, Chaponnier C, Brown RA (2002). Myofibroblasts and mechano-regulation of connective tissue remodelling. Nat Rev Mol Cell Biol.

[37] Burkhart RA, Peng Y, Norris ZA, Tholey RM, Talbott VA, Liang Q (2013). Mitoxantrone targets human ubiquitin-specific peptidase 11 (USP11) and is a potent inhibitor of pancreatic cancer cell survival. Mol Cancer Res.

[38] Chaudhary NI, Schnapp A, Park JE (2006). Pharmacologic differentiation of inflammation and fibrosis in the rat bleomycin model. Am J Respir Crit Care Med.

[39] Mulugeta S, Nureki S, Beers MF (2015). Lost after translation: insights from pulmonary surfactant for understanding the role of alveolar epithelial dysfunction and cellular quality control in fibrotic lung disease. Am J Physiol Lung Cell Mol Physiol.

[40] Ono S, Tanaka T, Ishida M, Kinoshita A, Fukuoka J, Takaki M (2011). Surfactant protein C G100S mutation causes familial pulmonary fibrosis in Japanese kindred. Eur Respir J.

[41] Thomas AQ, Lane K, Phillips J 3rd, Prince M, Markin C, Speer M (2002). Heterozygosity for a surfactant protein C gene mutation associated with usual interstitial pneumonitis and cellular nonspecific interstitial pneumonitis in one kindred. Am J Respir Crit Care Med.

[42] Nogee LM, Dunbar AE 3rd, Wert SE, Askin F, Hamvas A, Whitsett JA (2001). A mutation in the surfactant protein C gene associated with familial interstitial lung disease. N Engl J Med.

[43] Mulugeta S, Nguyen V, Russo SJ, Muniswamy M, Beers MF (2005). A surfactant protein C precursor protein BRICHOS domain mutation causes endoplasmic reticulum stress, proteasome dysfunction, and caspase 3 activation. Am J Respir Cell Mol Biol.

[44] Sitaraman S, Martin EP, Na CL, Zhao S, Green J, Deshmukh H (2021). Surfactant protein C mutation links postnatal type 2 cell dysfunction to adult disease. JCI Insight.

[45] Katzen J, Wagner BD, Venosa A, Kopp M, Tomer Y, Russo SJ (2019). An SFTPC BRICHOS mutant links epithelial ER stress and spontaneous lung fibrosis. JCI insight.

[46] Liu Y, Li Z, Xiao H, Xie B, He J, Song M (2023). USP13 Deficiency Impairs Autophagy and Facilitates Age-related Lung Fibrosis. Am J Respir Cell Mol Biol.

[47] Wang B, Li Y, Wang H, Zhao J, Zhao Y, Liu Z (2020). FOXO3a is stabilized by USP18-mediated de-ISGylation and inhibits TGF-β1-induced fibronectin expression. J Investig Med.

[48] Ge C, Huang M, Han Y, Shou C, Li D, Zhang Y (2024). Demethyleneberberine Alleviates Pulmonary Fibrosis through Disruption of USP11 Deubiquitinating GREM1. Pharmaceuticals.

[49] Jacko AM, Nan L, Li S, Tan J, Zhao J, Kass DJ (2016). De-ubiquitinating enzyme, USP11, promotes transforming growth factor β-1 signaling through stabilization of transforming growth factor β receptor II. Cell Death Dis.

[50] Tang Y, Yuan Q, Zhao C, Xu Y, Zhang Q, Wang L (2022). Targeting USP11 may alleviate radiation-induced pulmonary fibrosis by regulating endothelium tight junction. Int J Radiat Biol.

[51] Hill C, Jones MG, Davies DE, Wang Y (2019). Epithelial-mesenchymal transition contributes to pulmonary fibrosis via aberrant epithelial/fibroblastic cross-talk. J Lung Health Dis.

[52] Tanjore H, Cheng DS, Degryse AL, Zoz DF, Abdolrasulnia R, Lawson WE (2011). Alveolar epithelial cells undergo epithelial-to-mesenchymal transition in response to endoplasmic reticulum stress. J Biol Chem.

[53] Abou Taam R, Jaubert F, Emond S, Le Bourgeois M, Epaud R, Karila C (2009). Familial interstitial disease with I73T mutation: A mid- and long-term study. Pediatr Pulmonol.

[54] Crossno PF, Polosukhin VV, Blackwell TS, Johnson JE, Markin C, Moore PE (2010). Identification of early interstitial lung disease in an individual with genetic variations in ABCA3 and SFTPC. Chest.

[55] van Moorsel CH, van Oosterhout MF, Barlo NP, de Jong PA, van der Vis JJ, Ruven HJ (2010). Surfactant protein C mutations are the basis of a significant portion of adult familial pulmonary fibrosis in a dutch cohort. Am J Respir Crit Care Med.

[56] Mulugeta S, Maguire JA, Newitt JL, Russo SJ, Kotorashvili A, Beers MF (2007). Misfolded BRICHOS SP-C mutant proteins induce apoptosis via caspase-4- and cytochrome c-related mechanisms. Am J Physiol Lung Cell Mol Physiol.

[57] Tanjore H, Lawson WE, Blackwell TS (2013). Endoplasmic reticulum stress as a pro-fibrotic stimulus. Biochim Biophys Acta.

[58] Lawson WE, Cheng DS, Degryse AL, Tanjore H, Polosukhin VV, Xu XC (2011). Endoplasmic reticulum stress enhances fibrotic remodeling in the lungs. Proc Natl Acad Sci U S A.

[59] Kropski JA, Blackwell TS (2018). Endoplasmic reticulum stress in the pathogenesis of fibrotic disease. J Clin Invest.

[60] Rodriguez L, Tomer Y, Carson P, Dimopoulos T, Zhao M, Chavez K (2023). Chronic Expression of a Clinical SFTPC Mutation Causes Murine Lung Fibrosis with Idiopathic Pulmonary Fibrosis Features. Am J Respir Cell Mol Biol.

[61] Karapurkar JK, Colaco JC, Suresh B, Tyagi A, Woo SH, Jo WJ (2024). USP28 promotes tumorigenesis and cisplatin resistance by deubiquitinating MAST1 protein in cancer cells. Cell Mol Life Sci.

[62] Tyagi A, Kaushal K, Chandrasekaran AP, Sarodaya N, Das S, Park CH (2022). CRISPR/Cas9-based genome-wide screening for deubiquitinase subfamily identifies USP1 regulating MAST1-driven cisplatin-resistance in cancer cells. Theranostics.

[63] Yamamoto Y, Gotoh S, Korogi Y, Seki M, Konishi S, Ikeo S (2017). Long-term expansion of alveolar stem cells derived from human iPS cells in organoids. Nat Methods.

[64] Heo HR, Kim J, Kim WJ, Yang SR, Han SS, Lee SJ (2019). Human pluripotent stem cell-derived alveolar epithelial cells are alternatives for in vitro pulmotoxicity assessment. Sci Rep.

[65] Rasaei R, Kim E, Kim JY, Na S, Kim JH, Heo J (2020). Regulation of JAM2 Expression in the Lungs of Streptozotocin-Induced Diabetic Mice and Human Pluripotent Stem Cell-Derived Alveolar Organoids. Biomedicines.

[66] Heo HR, Hong SH (2021). Generation of macrophage containing alveolar organoids derived from human pluripotent stem cells for pulmonary fibrosis modeling and drug efficacy testing. Cell Biosci.

[67] Grinnell F (1999). Signal transduction pathways activated during fibroblast contraction of collagen matrices. Curr Top Pathol.

[68] Ngo P, Ramalingam P, Phillips JA, Furuta GT (2006). Collagen gel contraction assay. Methods Mol Biol.

