# Supplementary material for: Targeting USP11 counteracts SFTPCI73T-associated interstitial lung disease in hiPSCs-derived alveolar organoids and in vivo models
Source: Theranostics. 2025 Mar 19;15(10):4526–49. doi: 10.7150/thno.105994 (PMC11984411; doi:10.7150/thno.105994)
Supplement: Supplementary file 1 — Supplementary figures and tables. [file thnov15p4526s1.pdf]

Supplementary data

Targeting USP11 counteracts *SFTPC*^{I73T}-associated interstitial lung disease in hiPSCs-derived alveolar organoids and in vivo models

Janardhan Keshav Karapurkar[1,†], Sripriya Rajkumar[1,†], Ji-Hye Jung[2,†], Ji-Young Kim[2], Girish Birappa[1], D. A. Ayush Gowda[1], Jencia Carminha Colaco[1], Bharathi Suresh[1], Jung-Yun Choi[2], Sang Hyeon Woo[1], Won-Jun Jo[1], Jong-Hee Lee[3], Kye-Seong Kim[1,5]* Seok-Ho Hong[2,4]*, and Suresh Ramakrishna[1,5]*

[1]Graduate School of Biomedical Science and Engineering, Hanyang University, Seoul, 04763, South Korea.

[2]Department of Internal Medicine, College of Medicine, Kangwon National University, Chuncheon, Republic of Korea.

[3]National Primate Research Center (NPRC), Korea Research Institute of Bioscience and Biotechnology (KRIBB), Cheongju, 28116, South Korea.

[4]KW-Bio Co., Ltd, Chuncheon, Republic of Korea.

[5]College of Medicine, Hanyang University, Seoul, 04763, South Korea

Running title: Inhibition of USP11 alleviates SFTPC^{I73T}-associated interstitial lung disease

†These authors contributed equally.

***Corresponding authors**

SR (E-mail: suri28@hanyang.ac.kr, suresh.ramakris@gmail.com)

SH (E-mail: shhong@kangwon.ac.kr)

KS (E-mail: ks66kim@hanyang.ac.kr)

Supplementary Figures:

Figure S1. Confirmation of SFTPC^{I73T} mutation and validation.

Figure S2. Establishment of BEAS-2B cell lines stably expressing SFTPC^{I73T} protein.

Figure S3. The stabilizing effect of USP11 on both SFTPC and SFTPC^{I73T} protein levels was analyzed by increasing the concentrations of HA-USP11.

Figure S4. The stabilizing effect of USP11CS on both SFTPC and SFTPC^{I73T} protein levels.

Figure S5. Interactions between exogenous USP11 and SFTPC proteins.

Figure S6. The effect of silencing USP11 on the protein expression of SFTPC^{I73T} in the presence or absence of MG132.

Figure S7. USP11 deubiquitinates Myc-SFTPC^{I73T}.

Figure S8. The half-life of endogenous SFTPC and SFTPC^{I73T} in BEAS-2B cells.

Figure S9. The expression of USP11 in the presence of bleomycin in SFTPC and SFTPC^{I73T} cells.

Figure S10. The stabilizing effect of USP11 on SFTPC^{I73T} protein in BEAS-2B-SFTPC^{I73T} cells.

Figure S11. The graphical representation of the protein level of α-SMA and COL1A1.

Figure S12. Screening of hiPSCs carrying SFTPC^{I73T} mutation.

Figure S13. Validation of pluripotency status of hiPSCs carrying SFTPC^{I73T} mutation.

Figure S14. The expression of USP11 in USP11-depleted hiPSCs SFTPC-WT and hiPSCs carrying $SFTPC^{I73T}$ mutation.

Figure S15. qRT-PCR analysis of AOs using EMT markers.

Figure S16. TGF-β and BLM-induced fibrosis in AOs were analyzed by qRT PCR.

Figure S17. Body weight and survival rate of mice.

Figure S18. Immunohistochemical staining of mouse lung tissues.

Supplementary Tables

Table S1. Target sequences used for sgRNA plasmid construction.

Table S2. Target sequences used for shRNA plasmid construction.

Table S3. Oligonucleotide sequences used to get PCR amplicon for T7E1 assay.

Table S4. PCR amplicon and cleavage sizes after T7E1 assay.

Table S5. Oligonucleotide sequences used to get PCR amplicon for *ScaI* digestion.

Table S6. Primer sequences used for qRT-PCR**.**

Figure Legends

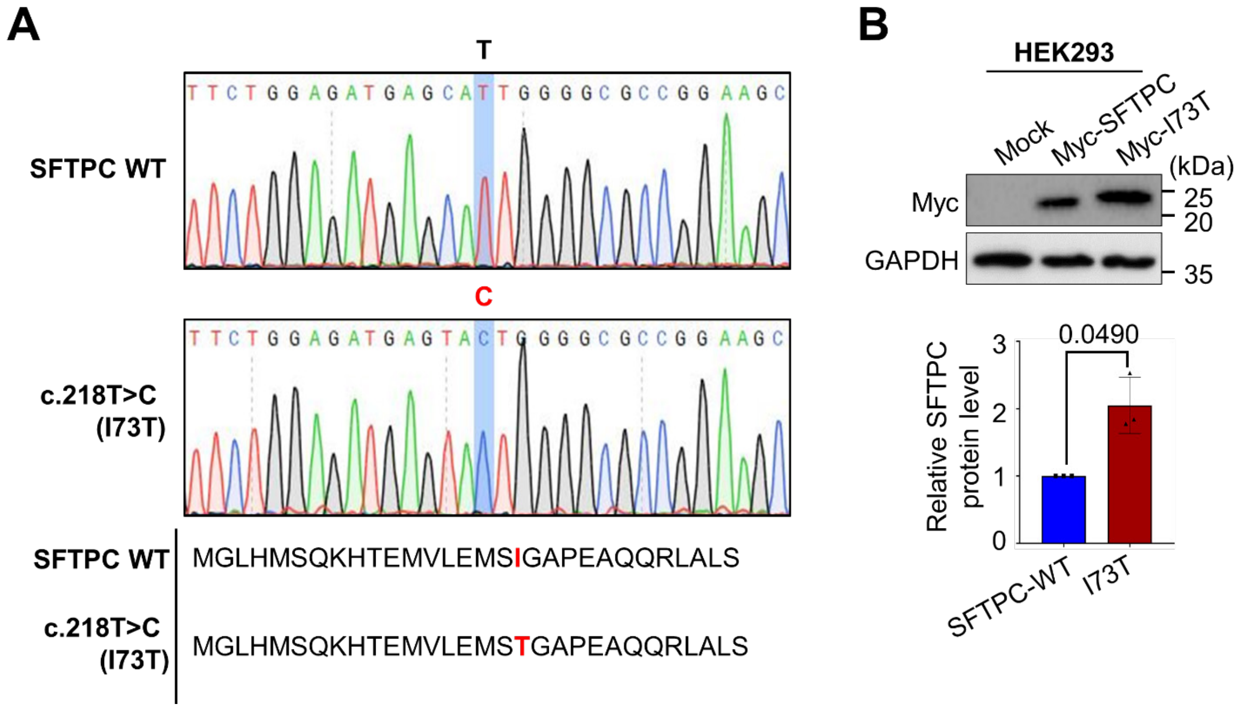

Figure S1. Confirmation of SFTPC^{I73T} mutation and validation. (A) Sanger sequencing analysis of SFTPC^{I73T} (T218C) missense mutations on the *SFTPC* gene. The amino acid substitutions for T218C and I73T are represented. The red font denotes the amino acid change from isoleucine (I) to threonine (T). **(B)** Western blot analysis of the basal level of SFTPC and SFTPC^{I73T} protein expression. Band intensity was estimated using ImageJ software, normalized to GAPDH, and represented graphically. Data are presented as the mean and standard deviation of three independent experiments (n = 3).

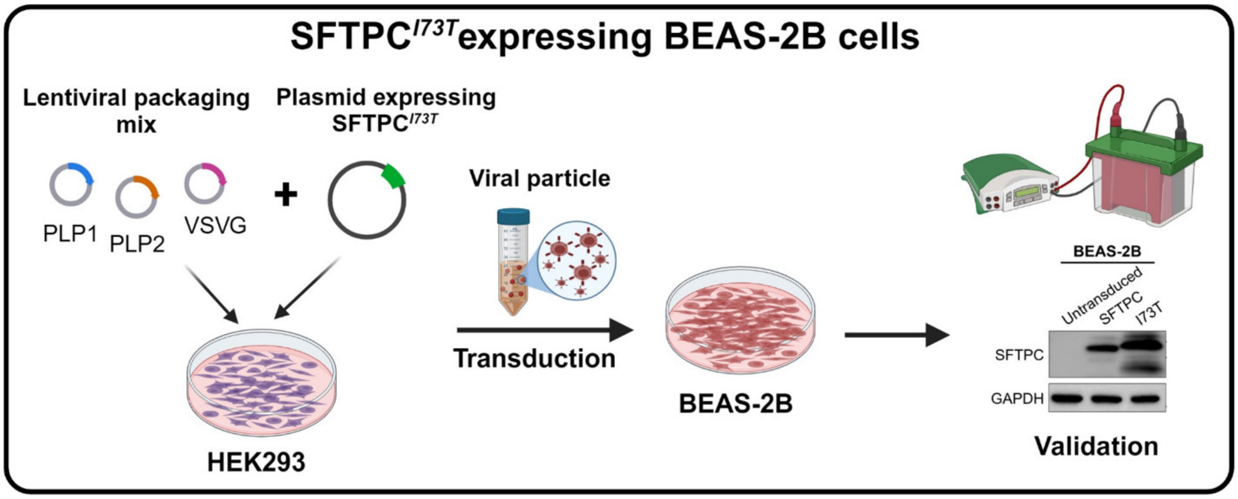

Figure S2. Establishment of BEAS-2B cell lines stably expressing SFTPC^{I73T} protein. HEK293 cells were transfected with a lentiviral vector encoding the *SFTPC*^{I73T} gene and packaging constructs. The viral particles were transduced into BEAS-2B cells, and the stable expression of the SFTPC^{I73T} protein was checked by western blot.

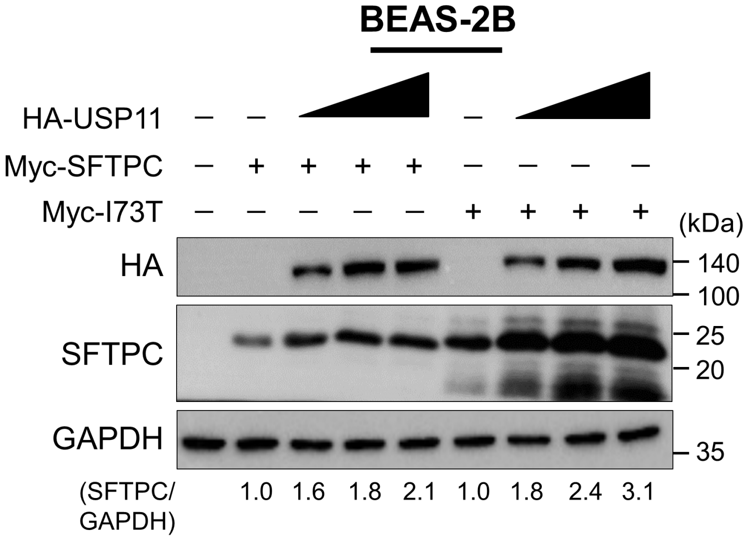

Figure S3. The stabilizing effect of USP11 on both SFTPC and SFTPC^{I73T} protein levels was analyzed by increasing the concentrations of HA-USP11.

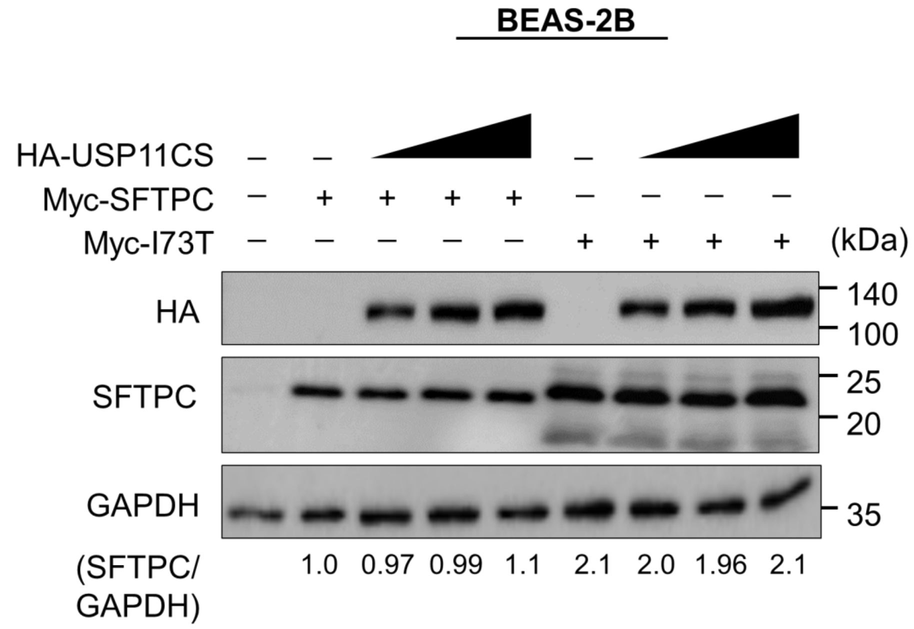

Figure S4. The stabilizing effect of USP11CS on both SFTPC and SFTPC^{I73T} protein levels was analyzed by western blotting.

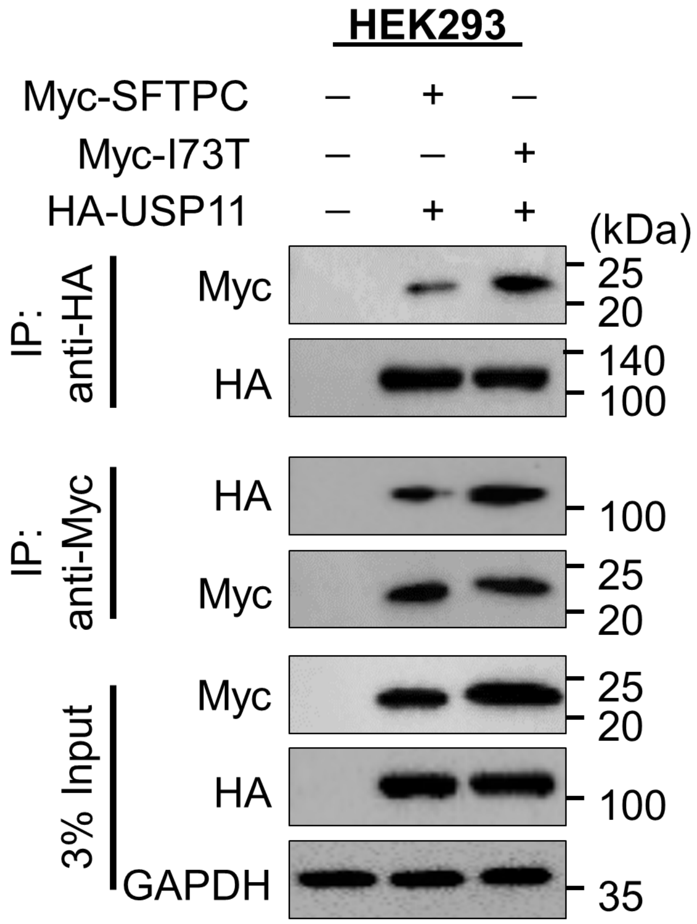

Figure S5. Interactions between exogenous USP11 and SFTPC proteins. Interaction was analyzed in HEK293. Cell lysates were immunoprecipitated and immunoblotted with the indicated antibodies.

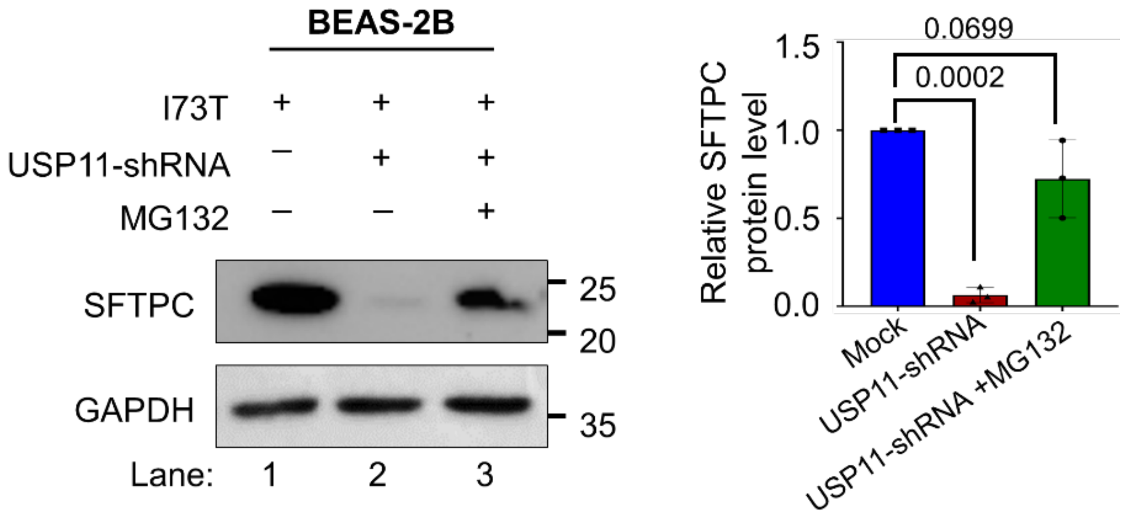

Figure S6. The effect of silencing USP11 on the protein expression of SFTPC^{I73T} in the presence or absence of MG132. Data are presented as the mean and standard deviation of three independent experiments (n = 3).

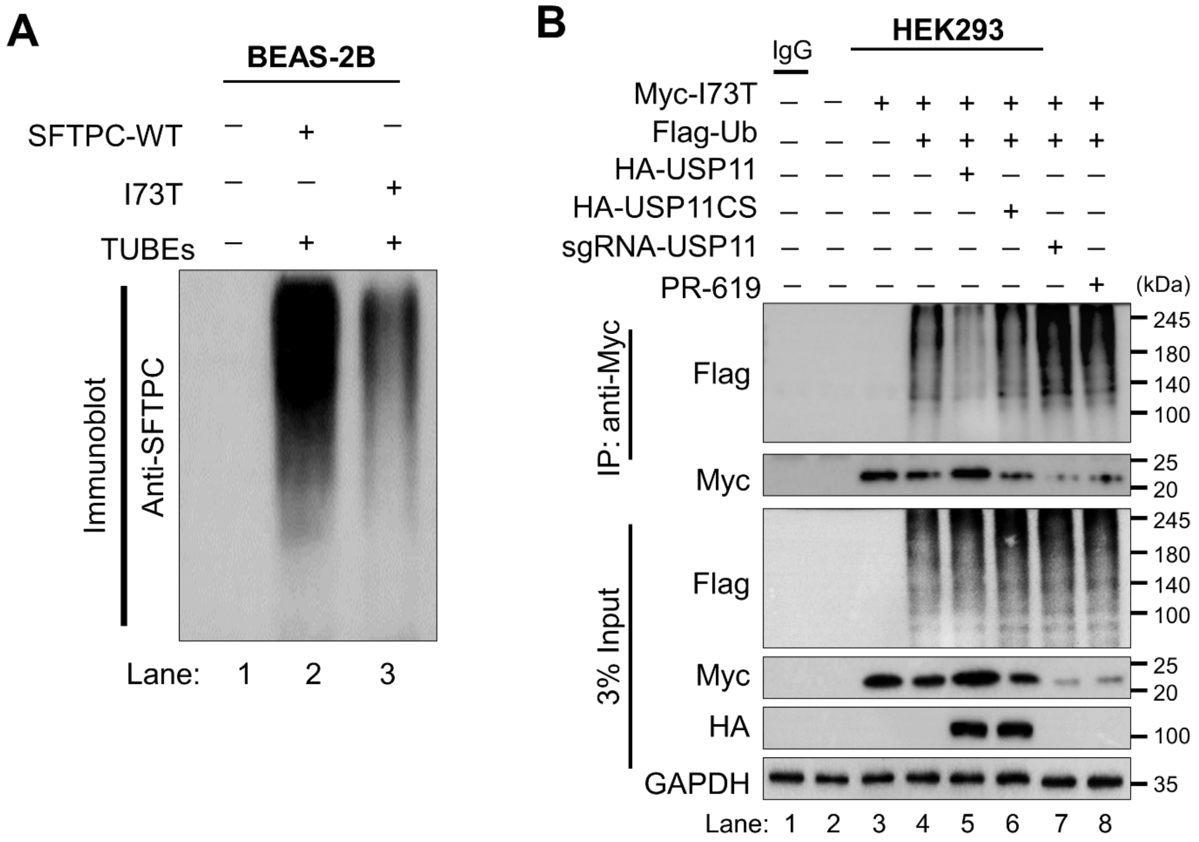

Figure S7. USP11 deubiquitinates Myc-SFTPC^{I73T}. (A) A TUBEs assay was performed to assess the ubiquitination status between the SFTPC and SFTPC^{I73T} proteins. Cell lysates were immunoprecipitated with TUBEs antibodies, followed by immunoblotting with the indicated antibodies. The cells were treated with MG132 for 6 h prior to harvest. **(B)** The ubiquitination and deubiquitination of ectopically expressed Myc-SFTPC^{I73T} were analyzed by transfecting HEK293 cells with Flag-Ub along with HA-USP11, HA-USP11CS, or sgRNA targeting USP11. IP was performed using the Myc antibody and immunoblotting with an anti-Flag antibody.

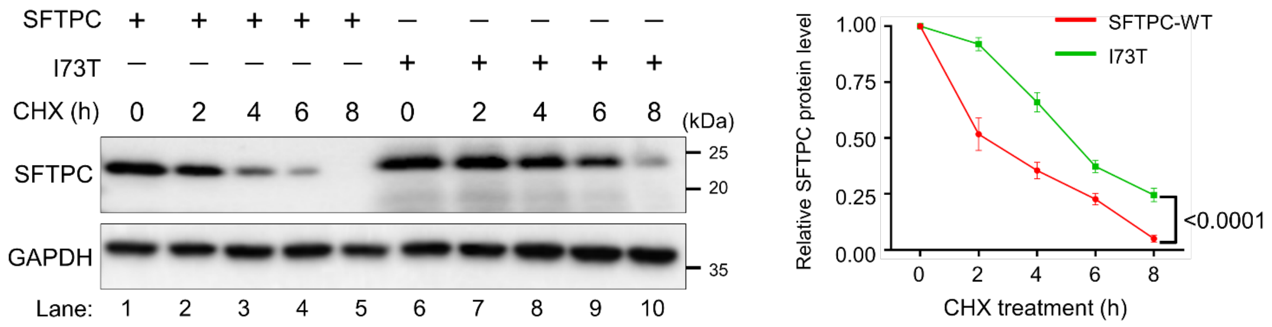

Figure S8. The half-life of endogenous SFTPC and SFTPC^{I73T} in BEAS-2B cells. CHX (250 μg/mL) was administered for the indicated time, and the cells were then harvested for western blotting with the indicated antibodies.

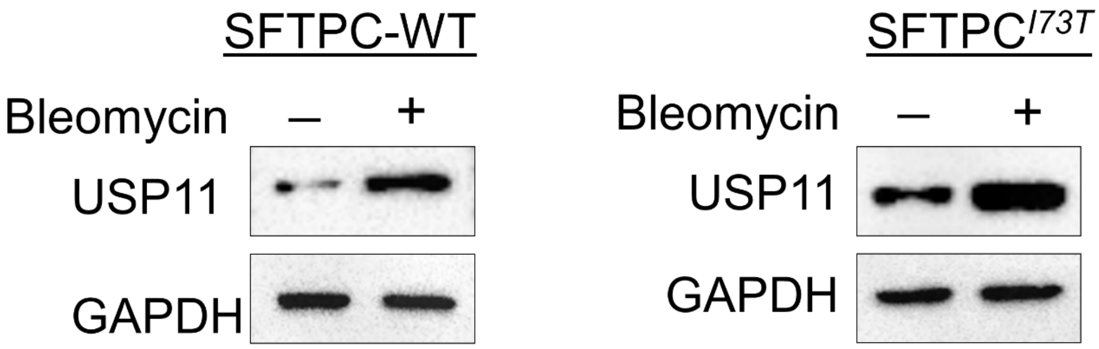

Figure S9. The expression of USP11 in the presence of bleomycin in BEAS-2B-SFTPC^{WT} and BEAS-2B-SFTPC^{I73T} cells.

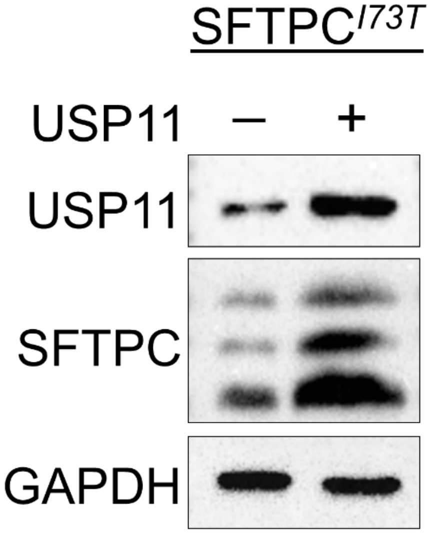

Figure S10. The stabilizing effect of USP11 on SFTPC^{I73T} protein in BEAS-2B-SFTPC^{I73T} cells.

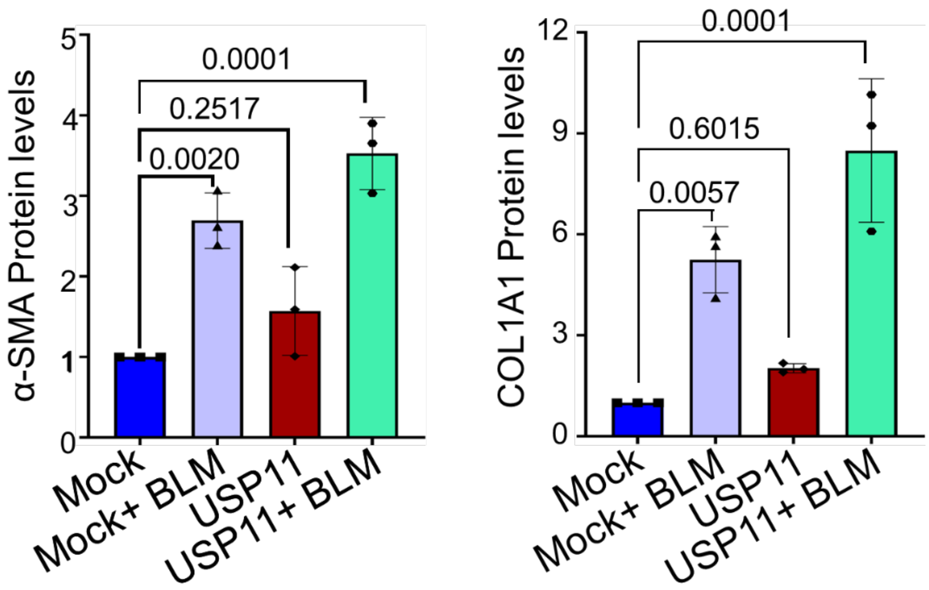

Figure S11. The graphical representation of the protein level of α-SMA and COL1A1. SFTPC^{I73T} cells treated with either BLM alone or a combination of BLM and USP11 transduction were subjected to western blot. Data are presented as the mean and standard deviation of three independent experiments (n = 3).

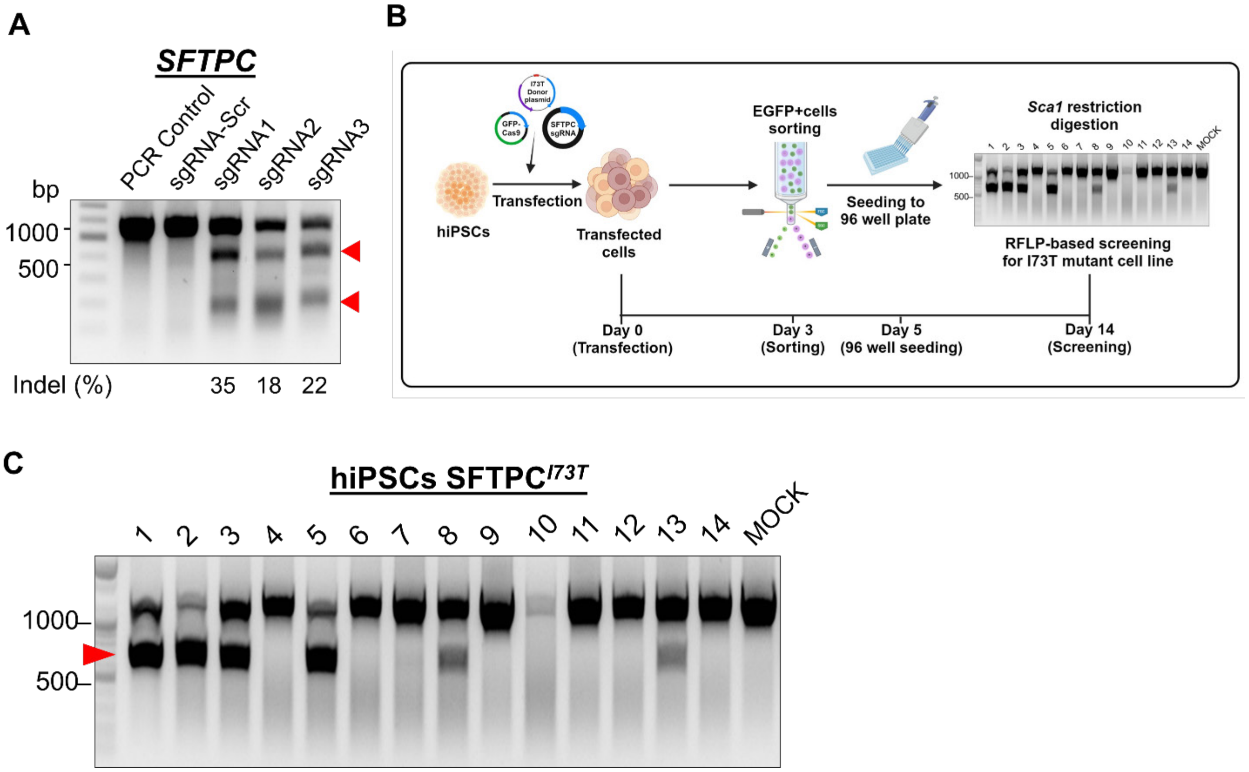

Figure S12. Screening of hiPSCs carrying SFTPC^{I73T} mutation. **(A)** The cleavage efficiency of sgRNAs targeting *SFTPC* gene by T7E1 assay. **(B)** Schematic representation showing how hiPSCs carrying the SFTPC^{I73T} mutation were generated. hiPSCs were transfected with Cas9-GFP, sgRNA1, and donor DNA with a homologous region of SFTPC with the I73T mutation. GFP-Cas9 expressing hiPSCs were sorted by flow cytometry and seeded onto 96-well plates. Screening was performed by RFLP method. **(C)** hiPSCs carrying SFTPC^{I73T} were screened by *ScaI* digestion.

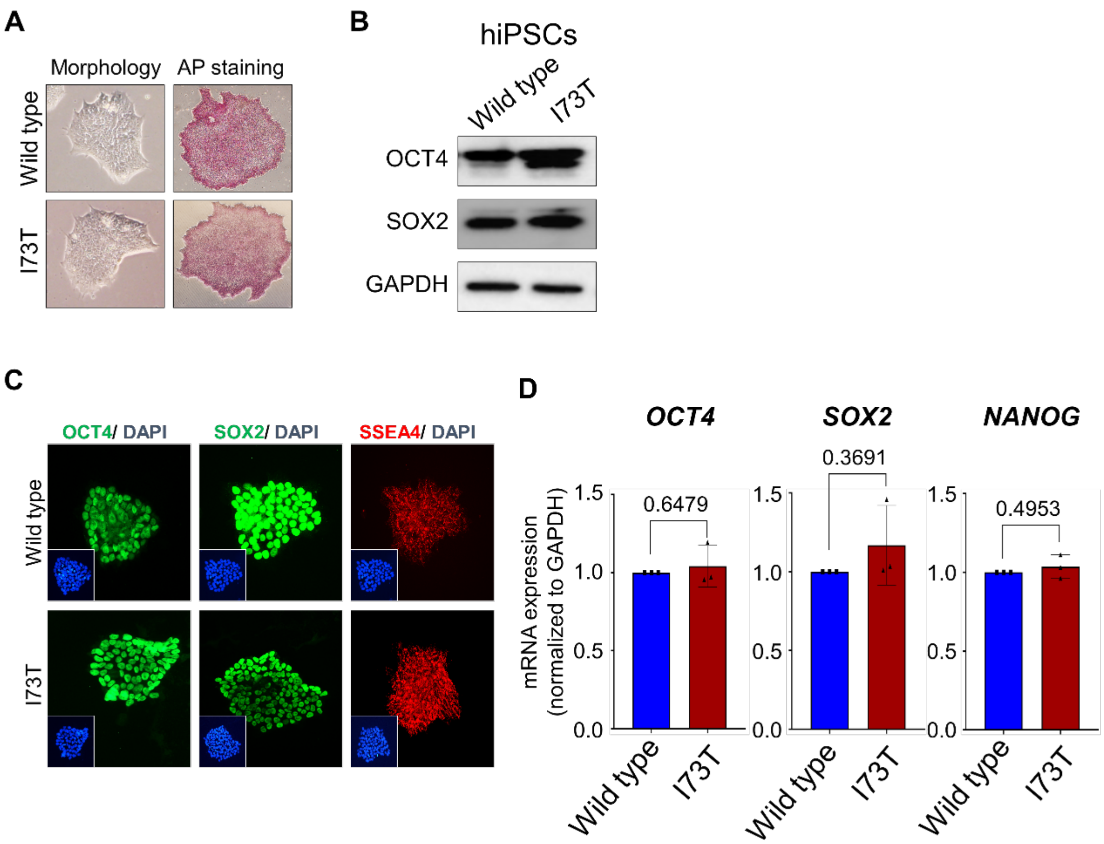

Figure S13. (A–D) Validation of the pluripotency behavior of hiPSCs carrying SFTPC^{I73T}. The cells were analyzed by **(A)** AP staining **(B)** western blot using OCT4 and SOX2 antibodies **(C)** immunostaining using the pluripotency markers OCT3/4, SOX2, and SSEA4, and **(D)** qRT-PCR using OCT4, SOX2 and NANOG primers. Data are presented as the mean and standard deviation of three independent experiments (n = 3).

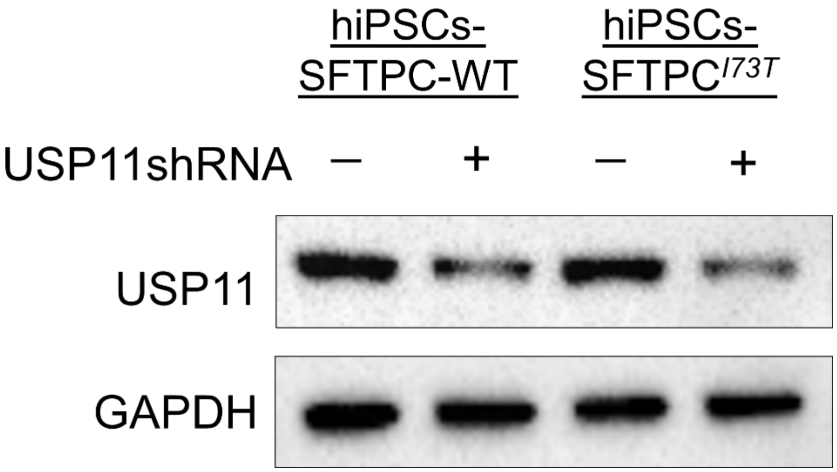

Figure S14. The expression of USP11 in USP11-depleted hiPSCs SFTPC-WT and hiPSCs carrying SFTPC^{I73T} mutation.

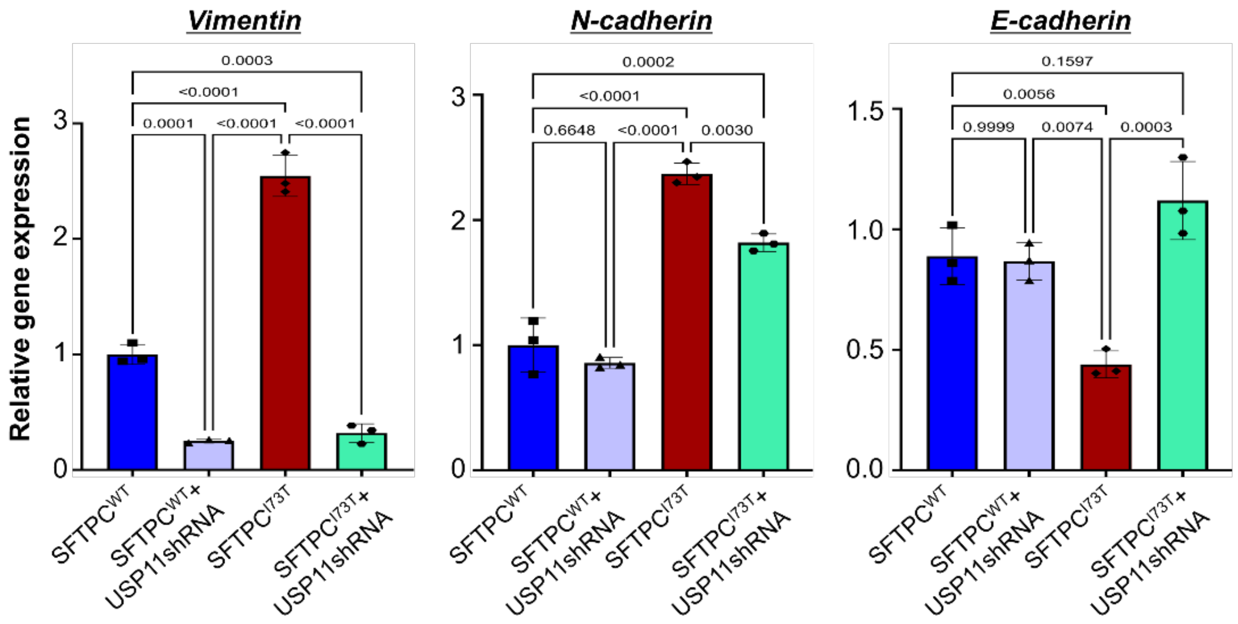

Figure S15. qRT-PCR analysis of AOs using EMT markers. The effect of USP11 depletion on SFTPC^{I73T}-mediated fibrosis in AOs was analyzed by qRT-PCR using EMT markers Vimentin, N-cadherin and E-cadherin. Data are presented as the mean and standard deviation of three independent experiments (n = 3).

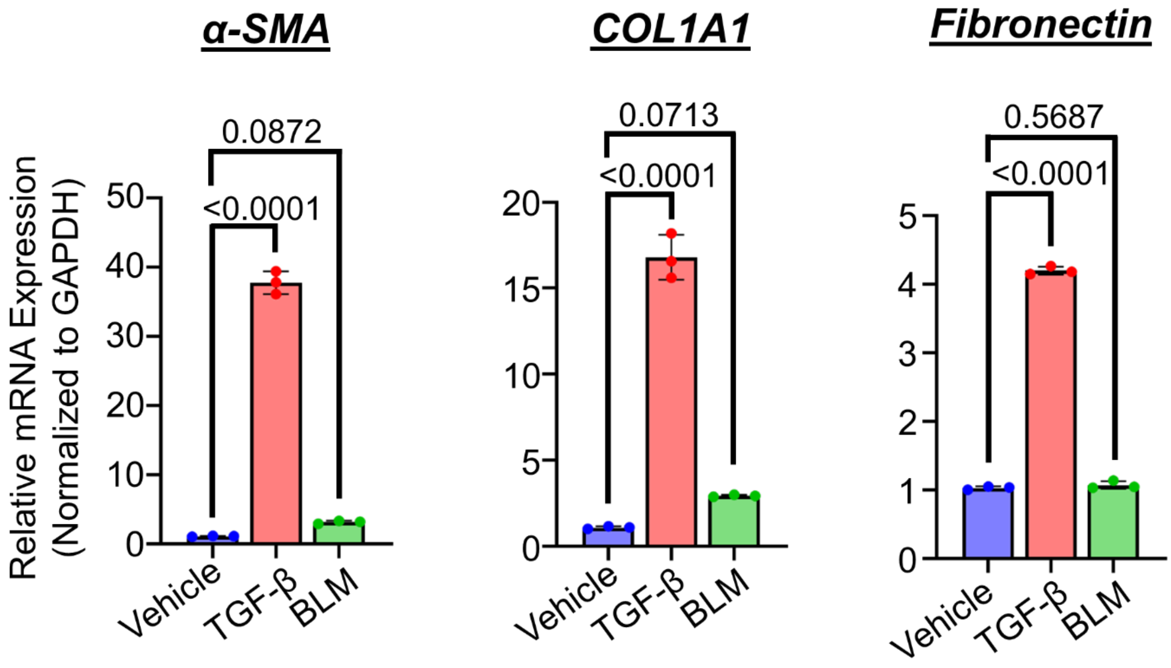

Figure S16. TGF-β and BLM-induced fibrosis in AOs were analyzed by qRT PCR.

TGF-β at the concentration of 25 ng/mL and BLM at the concentration of 3μg/mL was used to induce fibrosis in AOs. Fibrotic markers COL1A1, α-SMA and Fibronectin were analyzed by qRT PCR.

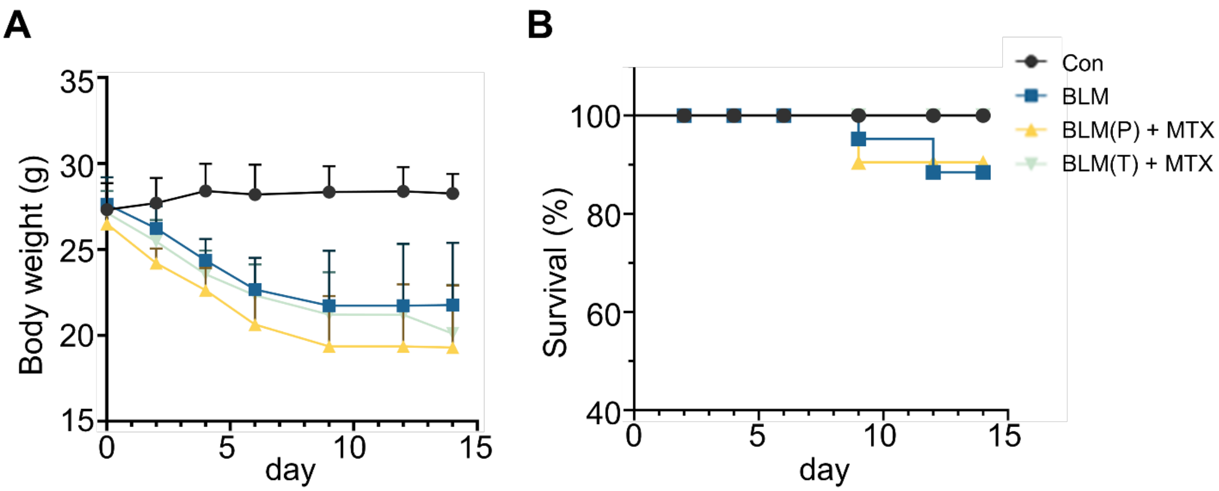

Figure S17. Body weight and survival rate of mice. **(A)** The recorded body weight of mice used in experimental groups. **(B)** The survival rate of the mice from different experimental groups. Data are presented as the mean and standard deviation.

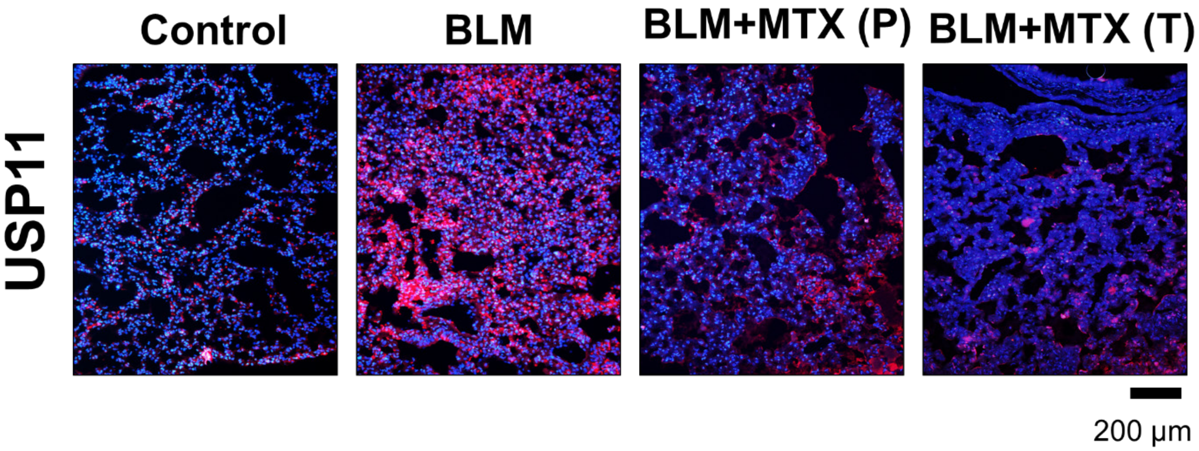

Figure S18. Immunohistochemical staining of mouse lung tissues. Lung tissues from the BLM-induced mice in both the preventive group (P) and therapeutic group (T) were sectioned and paraffin embedded. Immunohistochemical staining using USP11 antibody.

Table S1. Target sequences used for sgRNA plasmid construction.

Gene	Species	sgRNA	Sequence (5’ to 3’)
USP11	*Homo sapiens*	sgRNA1	GCGTTGGCTGTAGAAGAGAA
		sgRNA2	GCACTGGTATAAGCAGTGGG
SFTPC	*Homo sapiens*	sgRNA1	CAGGTTCTGGAGATGAGCAT
		sgRNA2	GGTTCTGGAGATGAGCATTG
		sgRNA3	GGAGATGAGCATTGGGGCGC

Table S2. Target sequences used for shRNA plasmid construction.

Gene	Species	shRNA	Sequence (5’ to 3’)
USP11	*Homo sapiens*	shRNA1	CCGTGATGATATCTTCGTCTA
		shRNA2	CGGCACAATGATTTGGGCAAA

Table S3. Oligonucleotide sequences used to get PCR amplicon for T7E1 assay.

Gene		sgRNA		Direction	Sequence (5' to 3')
USP11	Homo sapiens	sgRNA1	I PCR	FP	TCAGCGTCCCCATTGTTACC
				RP	CCTTTTCCAGACGTTGCGAT
			II PCR	FP1	GGGTCTCTGGAGGTGGAAAC
				RP1	CCAGCCTCACCAGCTTTCGC
		sgRNA2	I PCR	FP	AAATTAGCTGCAGATGGCACA
				RP	CCCCATCCATTATACCTTGCGT
			II PCR	FP1	TGTTGGTTTGTTTGCCATTG
SFTPC	Homo sapiens	sgRNA1, sgRNA2 and sgRNA3	I PCR	FP	AGCCAGAAACACACGGAGAT
				RP	GATGGATGTGGATGAAGTGGC
			II PCR	FP1	AGCCAGAAACACACGGAGAT
				RP1	AGCAGAGCCTTGTCATTGGTC

Table S4. PCR amplicon and cleavage sizes after T7E1 assay

Gene	sgRNA	PCR size (bp)	Cleavage size (bp)
USP11	sgRNA1	579	378+201
	sgRNA2	681	286+395
SFTPC	sgRNA1	541	379+162
	sgRNA2		384+157
	sgRNA3		387+154

Table S5. Oligonucleotide sequences used to get PCR amplicon for *ScaI* digestion.

Gene	Primer	Primer sequence	Amplicon size (bp)
SFTPC	*SFTPC*^{I73T}-FP	GTTAGAATCCAGGCCACCTCC	1372 bp
	SFTPC^{I73T}-RP	TCAGAAGCCACCAGGTAGTGAG	
	SFTPC^{I73T}-FP1	CCTTCCCTGTCCATCCATCG	1250 bp
	SFTPC^{I73T}-RP1	TCGTGCAGGGGAATAGGAGA	

Table S6. Primer sequences used for qRT-PCR

Gene		Sequence (5' to 3')
OCT4	FP	TAGCATTGAGAACCGTGTGAG
	RP	ACTTGATCTTTTGCCCTTCTGG
SOX2	FP	GAGCTTTGCAGGAAGTTTGC
	RP	GCAAGAAGCCTCTCCTTGAA
NANOG	FP	ACCTTGGCTGCCGTCTCTGG
	RP	AGCAAAGCCTCCCAATCCCAAACA
COL1A1	FP	AAGGGTGAGACAGGCGAACA
	RP	GACCCTGGAGGCCAGAGAAG
Fibronectin	FP	ACAACACCGAGGTGACTGAGAC
	RP	GGACACAACGATGCTTCCTGAG
α-SMA	FP	GACGAAGCACAGAGCAAAAG
	RP	AGTTGGTGATGATGCCATGT
Vimentin	FP	CCAGGCAAAGCAGGAGTC
	RP	CGAAGGTGACGAGCCATT
N-cadherin	FP	GGTGGAGGAGAAGAAGACCAG
	RP	GGCATCAGGCTCCACAGT
E-cadherin	FP	CTGAGAACGAGGCTAACG
	RP	TTCACATCCAGCACATCC

SFTPB	FP	GCCATACCACAGGCAATGCT
	RP	TGCTGCTCCACAAATTGCTT
SFTPC	FP	CCTTCTTATCGTGGTGGTGGT
	RP	TCTCCGTGTGTTTCTGGCTCAT
GAPDH	FP	TGCACCACCAACTGCTTAGC
	RP	GGCATGGACTGTGGTCATGAG
SFTPA	FP	AAGCAGCTGGAGGCTCTGT
	RP	CCATCAAGATGAGGGTGAGG
NKX2.1	*FP*	AGCACACGACTCCGTTCTC
	RP	GCCCACTTTCTTGTAGCTTTCC
